# DeePMD-kit v2: A software package for deep potential models

**DOI:** 10.1063/5.0155600

**Published:** 2023-08-01

**Authors:** Jinzhe Zeng, Duo Zhang, Denghui Lu, Pinghui Mo, Zeyu Li, Yixiao Chen, Marián Rynik, Li’ang Huang, Ziyao Li, Shaochen Shi, Yingze Wang, Haotian Ye, Ping Tuo, Jiabin Yang, Ye Ding, Yifan Li, Davide Tisi, Qiyu Zeng, Han Bao, Yu Xia, Jiameng Huang, Koki Muraoka, Yibo Wang, Junhan Chang, Fengbo Yuan, Sigbjørn Løland Bore, Chun Cai, Yinnian Lin, Bo Wang, Jiayan Xu, Jia-Xin Zhu, Chenxing Luo, Yuzhi Zhang, Rhys E. A. Goodall, Wenshuo Liang, Anurag Kumar Singh, Sikai Yao, Jingchao Zhang, Renata Wentzcovitch, Jiequn Han, Jie Liu, Weile Jia, Darrin M. York, Weinan E, Roberto Car, Linfeng Zhang, Han Wang

**Affiliations:** 1 Laboratory for Biomolecular Simulation Research, Institute for Quantitative Biomedicine and Department of Chemistry and Chemical Biology, Rutgers University, Piscataway, New Jersey 08854, USA; 2 AI for Science Institute, Beijing 100080, People’s Republic of China; 3 DP Technology, Beijing 100080, People’s Republic of China; 4 Academy for Advanced Interdisciplinary Studies, Peking University, Beijing 100871, People’s Republic of China; 5 HEDPS, CAPT, College of Engineering, Peking University, Beijing 100871, People’s Republic of China; 6 College of Electrical and Information Engineering, Hunan University, Changsha, People’s Republic of China; 7 Yuanpei College, Peking University, Beijing 100871, People’s Republic of China; 8 Program in Applied and Computational Mathematics, Princeton University, Princeton, New Jersey 08540, USA; 9 Department of Experimental Physics, Comenius University, Mlynská Dolina F2, 842 48 Bratislava, Slovakia; 10 Center for Quantum Information, Institute for Interdisciplinary Information Sciences, Tsinghua University, Beijing 100084, People’s Republic of China; 11 Center for Data Science, Peking University, Beijing 100871, People’s Republic of China; 12 ByteDance Research, Zhonghang Plaza, No. 43, North 3rd Ring West Road, Haidian District, Beijing, People’s Republic of China; 13 College of Chemistry and Molecular Engineering, Peking University, Beijing 100871, People’s Republic of China; 14 Baidu, Inc., Beijing, People’s Republic of China; 15 Key Laboratory of Structural Biology of Zhejiang Province, School of Life Sciences, Westlake University, Hangzhou, Zhejiang, People’s Republic of China; 16 Westlake AI Therapeutics Lab, Westlake Laboratory of Life Sciences and Biomedicine, Hangzhou, Zhejiang, People’s Republic of China; 17 Department of Chemistry, Princeton University, Princeton, New Jersey 08544, USA; 18 SISSA, Scuola Internazionale Superiore di Studi Avanzati, 34136 Trieste, Italy; 19 Laboratory of Computational Science and Modeling, Institute of Materials, École Polytechnique Fédérale de Lausanne, 1015 Lausanne, Switzerland; 20 Department of Physics, National University of Defense Technology, Changsha, Hunan 410073, People’s Republic of China; 21 State Key Lab of Processors, Institute of Computing Technology, Chinese Academy of Sciences, Beijing, People’s Republic of China; 22 University of Chinese Academy of Sciences, Beijing, People’s Republic of China; 23 School of Electronics Engineering and Computer Science, Peking University, Beijing 100871, People’s Republic of China; 24 Department of Chemical System Engineering, The University of Tokyo, 7-3-1 Hongo, Bunkyo-ku, Tokyo 113-8656, Japan; 25 Hylleraas Centre for Quantum Molecular Sciences and Department of Chemistry, University of Oslo, P.O. Box 1033 Blindern, 0315 Oslo, Norway; 26 Wangxuan Institute of Computer Technology, Peking University, Beijing 100871, People’s Republic of China; 27 Shanghai Engineering Research Center of Molecular Therapeutics and New Drug Development, Shanghai Key Laboratory of Green Chemistry and Chemical Process, School of Chemistry and Molecular Engineering, East China Normal University, Shanghai 200062, People’s Republic of China; 28 School of Chemistry and Chemical Engineering, Queen’s University Belfast, Belfast BT9 5AG, United Kingdom; 29 State Key Laboratory of Physical Chemistry of Solid Surfaces, iChEM, College of Chemistry and Chemical Engineering, Xiamen University, Xiamen 361005, People’s Republic of China; 30 Department of Applied Physics and Applied Mathematics, Columbia University, New York, New York 10027, USA; 31 Independent Researcher, London, United Kingdom; 32 Department of Data Science, Indian Institute of Technology, Palakkad, Kerala, India; 33 NVIDIA AI Technology Center (NVAITC), Santa Clara, California 95051, USA; 34 Department of Earth and Environmental Sciences, Columbia University, New York, New York 10027, USA; 35 Center for Computational Mathematics, Flatiron Institute, New York, New York 10010, USA; 36 Center for Machine Learning Research and School of Mathematical Sciences, Peking University, Beijing 100871, People’s Republic of China; 37 Laboratory of Computational Physics, Institute of Applied Physics and Computational Mathematics, Fenghao East Road 2, Beijing 100094, People’s Republic of China

## Abstract

DeePMD-kit is a powerful open-source software package that facilitates molecular dynamics simulations using machine learning potentials known as Deep Potential (DP) models. This package, which was released in 2017, has been widely used in the fields of physics, chemistry, biology, and material science for studying atomistic systems. The current version of DeePMD-kit offers numerous advanced features, such as DeepPot-SE, attention-based and hybrid descriptors, the ability to fit tensile properties, type embedding, model deviation, DP-range correction, DP long range, graphics processing unit support for customized operators, model compression, non-von Neumann molecular dynamics, and improved usability, including documentation, compiled binary packages, graphical user interfaces, and application programming interfaces. This article presents an overview of the current major version of the DeePMD-kit package, highlighting its features and technical details. Additionally, this article presents a comprehensive procedure for conducting molecular dynamics as a representative application, benchmarks the accuracy and efficiency of different models, and discusses ongoing developments.

## I. INTRODUCTION

In recent years, the increasing popularity of machine learning potentials (MLPs) has revolutionized molecular dynamics (MD) simulations across various fields, including neural network potentials (NNPs),^1–19^ message passing models,^7,20–24^ and other machine learning models.^25–28^ Numerous software packages have been developed to support the use of MLPs.^13,29–40^ One of the main reasons for the widespread adoption of MLPs is their exceptional speed and accuracy, which outperform traditional molecular mechanics (MM) and *ab initio* quantum mechanics (QM) methods.^41,42^ As a result, MLP-powered MD simulations have become ubiquitous in the field and are increasingly recognized as a valuable tool for studying atomistic systems.^43–49^

The DeePMD-kit is an open-source software package that facilitates molecular dynamics (MD) simulations using neural network potentials. The package was first released in 2017^29^ and has since undergone rapid development with contributions from many developers. The DeePMD-kit implements a series of MLP models known as Deep Potential (DP) models,^9,10,50–54^ which have been widely adopted in the fields of physics, chemistry, biology, and material science for studying a broad range of atomistic systems. These systems include metallic materials,^55^ non-metallic inorganic materials,^56–60^ water,^61–71^ organic systems,^10,72^ solutions,^52,73–76^ gas-phase systems,^77–80^ macromolecular systems,^81,82^ and interfaces.^83–87^ Furthermore, the DeePMD-kit is capable of simulating systems containing almost all Periodic Table elements,^51^ operating under a wide range of temperature and pressure,^88^ and can handle drug-like molecules,^72,89^ ions,^73,76^ transition states,^75,77^ and excited states.^90^ As a result, the DeePMD-kit is a powerful and versatile tool that can be used to simulate a wide range of atomistic systems. Here, we present three exemplary instances that highlight its diverse applications.

Theoretical investigation of the water phase diagram poses a significant challenge due to the requirement for a highly accurate model of water interatomic interactions.^91,92^ Consequently, it serves as an exceptionally stringent test for the model’s accuracy and provides a means to validate the software implementation necessary for molecular dynamics simulations used in phase diagram calculations.^92^ Zhang *et al.*^88^ utilized DeePMD-kit to construct a deep potential model for the water system, covering a range of thermodynamic states from 0 to 2400 K and 0–50 GPa. The model was trained on density functional theory (DFT) data generated using the SCAN approximation of the exchange–correlation functional and exhibited consistent accuracy [with an Root mean square error (RMSE) of less than 1 meV/H2O] within the relevant thermodynamic range. Moreover, it accurately predicted fluid, molecular, and ionic phases and all stable ice polymorphs within the range, except for phases III and XV. The study extensively investigated the two first-order phase transitions from ice VII to VII” and VII” to ionic fluid and the atomistic mechanism of proton diffusion, leveraging the model’s capability and high accuracy in predicting water molecule ionization.

Another challenging area is condensed-phase MD simulations, as long-range interactions are critical for modeling heterogeneous systems in the condensed phase. Electrostatic interactions are not only the longest but also are well-understood, and linear-scaling methods exist for their efficient computation at the point charge,^93^ multipole,^94,95^ and quantum mechanical^96,97^ levels. Fast semiempirical quantum mechanical methods can be developed^98,99^ that can accurately and efficiently model charge densities and many-body effects in the long-range but may still lack quantitative accuracy in the mid-range (typically less than 8 Å). This limits the predictive capability of the methods in condensed-phase simulations. Zeng *et al.*^52^ created a new Δ-MLP method called Deep Potential-Range correction (DPRc) to integrate with combined quantum mechanical/molecular mechanical (QM/MM) potentials, which corrects the potential energy from a fast, linear-scaling low-level semiempirical QM/MM theory to a high-level *ab initio* QM/MM theory. Unlike many of the emerging Δ-MLPs that correct internal QM energy and forces, the DPRc model corrects both the QM–QM and QM–MM interactions of a QM/MM calculation in a manner that conserves energy as MM atoms enter (or leave) the vicinity of the QM region. This enables the model to be easily integrated as a mid-ranged correction to the potential energy within molecular simulation software that uses non-bonded lists, i.e., for each atom, a list of other atoms within a fixed cut-off distance (typically 8–12 Å). The trained DPRc model with a 6 Å range-correction was applied to simulate RNA 2′-O-transphosphorylation reactions in solution in long timescales^75^ and obtain better free energy estimates with the help of the generalization of the weighted thermodynamic perturbation (gwTP) method.^100^ Very recently, Zeng *et al.*^72^ have trained a Δ-MLP correction model called Quantum Deep Potential Interaction (QD*π*) for drug-like molecules, including tautomeric forms and protonation states, which was found to be superior to other semiempirical methods and pure MLP models.^89^

The third important application is large-scale reactive MD simulations over a nanosecond time scale, which enable the construction of interwoven reaction networks for complex reactive systems^101^ instead of focusing on studying a single reaction. These simulations require the potential energy model to be accurate and computationally efficient, covering the chemical space of possible reactions. Zeng *et al.*^77^ introduced a deep potential model for simulating 1 ns methane combustion reactions and identified 798 different chemical reactions in both space and time using the ReacNetGenerator package.^102^ The concurrent learning procedure^103^ was adopted and proved crucial in exploring known and unknown chemical space during the complex reaction process. Subsequent work conducted by the research team extended these simulations to more complex reactive systems, including linear alkane pyrolysis,^78^ decomposition of explosive,^79,104^ and the growth of polycyclic aromatic hydrocarbon.^80^

Compared to its initial release,^29^ DeePMD-kit has evolved significantly, with the current version (v2.2.1) offering an extensive range of features. These include DeepPot-SE, attention-based, and hybrid descriptors,^10,50,51,53^ the ability to fit tensorial properties,^105,106^ type embedding, model deviation,^103,107^ Deep Potential-Range Correction (DPRc),^52,75^ Deep Potential Long Range (DPLR),^53^ graphics processing unit (GPU) support for customized operators,^108^ model compression,^109^ non-von Neumann molecular dynamics (NVNMD),^110^ and various usability improvements, such as documentation, compiled binary packages, graphical user interfaces (GUIs), and application programming interfaces (APIs). This article provides an overview of the current major version of the DeePMD-kit, highlighting its features and technical details, presenting a comprehensive procedure for conducting molecular dynamics as a representative application, benchmarking the accuracy and efficiency of different models, and discussing ongoing developments.

## II. FEATURES

**FIG. 1.**
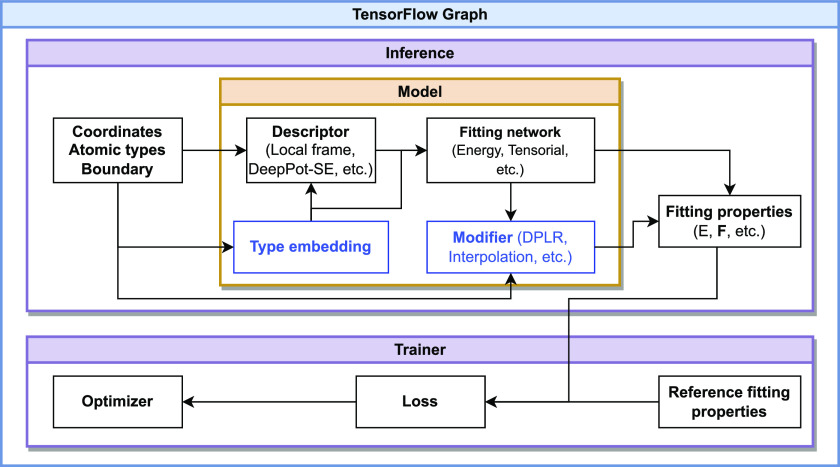
The components of the DeePMD-kit package. The direction of the arrow indicates the dependency between the components. The blue box represents an optional component.

In this section, we introduce features from the perspective of components (shown in Fig. 1). A component represents units of computation. It is organized as a Python class inside the package, and a corresponding TensorFlow static graph will be created at runtime.

### A. Models

A Deep Potential (DP) model, denoted by M, can be generally represented as

$$y_{i}=M\left(x_{i},{\left\{x_{\;j}\right\}}_{j\in n\left(i\right)};\theta \right)=F\left(D\left(x_{i},{\left\{x_{\;j}\right\}}_{j\in n\left(i\right)};\theta_{d}\right);\theta_{\;f},\;\right),$$ (1)

where ***y***_*i*_ is the fitting properties, F is the fitting network (introduced in Sec. II A 3), and D is the descriptor (introduced in Sec. II A 2). ***x*** = (***r***_*i*_, *α*_*i*_), with ***r***_*i*_ being the Cartesian coordinates and *α*_*i*_ being the chemical species, denotes the degrees of freedom of the atom *i*. The indices of the neighboring atoms (i.e., atoms within a certain cutoff radius) of atom *i* are given by the notation *n*(*i*). Note that the Cartesian coordinates can be either under the periodic boundary condition (PBC) or in vacuum (under the open boundary condition). The network parameters are denoted by ***θ*** = {***θ***_*d*_, ***θ***_*f*_}, where ***θ***_*d*_ and ***θ***_*f*_ yield the network parameters of the descriptor (if any) and those of the fitting network, respectively. From Eq. (1), one may compute the global property of the system by

$$y=\overset{N}{\underset{i=1}{\sum }}y_{i},$$ (2)

where *N* is the number of atoms in a frame. For example, if *y*_*i*_ represents the potential energy contribution of atom *i*, then *y* gives the total potential energy of the frame. In the following text, *N*_*c*_ is the expected maximum number of neighboring atoms, which is the same constant for all atoms over all frames. A matrix with a dimension of *N*_*c*_ will be padded if the number of neighboring atoms is less than *N*_*c*_.

#### 1. Neural networks

A neural network (NN) function N is the composition of multiple layers $L^{\left(i\right)}$,

$$N=L^{\left(n\right)}◦L^{\left(n-1\right)}◦\cdots ◦L^{\left(1\right)}.$$ (3)

In the DeePMD-kit package, a layer L may be one of the following forms depending on whether a ResNet^111^ is used and the number of nodes:

$$y=L\left(x;w,b\right)=\left\{\;,\begin{array}{cc} \hat{w}⊙\phi \left(x^{T}w+b\right)+x,\; & \;\text{ResNet and }N_{2}=N_{1},\\ \hat{w}⊙\phi \left(x^{T}w+b\right)+\left\{x,x\right\},\; & \;\text{ResNet and }N_{2}=2N_{1},\\ \hat{w}⊙\phi \left(x^{T}w+b\right),\; & \text{otherwise}, \end{array}\right.$$ (4)

where $x\in R^{N_{1}}$ is the input vector and $y\in R^{N_{2}}$ is the output vector. $w\in R^{N_{1}\times N_{2}}$ and $b\in R^{N_{2}}$ are weights and biases, respectively, both of which are trainable. $\hat{w}\in R^{N_{2}}$ can be either a trainable vector, which represents the “timestep” in the skip connection, or a vector of all ones **1** = {1, 1, …, 1}, which disables the time step. ***ϕ*** is the activation function. In theory, the activation function can be any form, and the following functions are provided in the DeePMD-kit package: hyperbolic tangent (tanh), rectified linear unit (ReLU),^112^ ReLU6, softplus,^113^ sigmoid, Gaussian error linear unit (GELU),^114^ and identity. Among these activation functions, ReLU and ReLU6 are not continuous in the first-order derivative, and others are continuous up to the second-order derivative.

#### 2. Descriptors

DeePMD-kit supports multiple atomic descriptors, including the local frame descriptor, the two-body and three-body embedding DeepPot-SE descriptor, the attention-based descriptor, and the hybrid descriptor that is defined as a combination of multiple descriptors. In the following text, we use $D^{\;i}=D\left(x_{i},{\left\{x_{\;j}\right\}}_{j\in n\left(i\right)};\theta_{d}\right)$ to represent the atomic descriptor of the atom *i*.

##### a. Local frame.

The local frame descriptor $D^{\;i}\in R^{N_{c}\times \left\{1,4\right\}}$ (sometimes simply called the DPMD model), which is the first version of the DP descriptor,^9^ is constructed by using either full information or radial-only information,

$${\left(D^{\;i}\right)}_{j}=\left\{\;,\begin{array}{cc} \left\{\;,\begin{array}{cccc} \;\frac{1}{r_{i\;j}} & \frac{x_{i\;j}}{r_{i\;j}} & \frac{y_{i\;j}}{r_{i\;j}} & \frac{z_{i\;j}}{r_{i\;j}} \end{array},\;\right\},\; & \text{full},\\ \left\{\;,\begin{array}{c} \frac{1}{r_{i\;j}} \end{array},\;\right\},\; & \text{radial}-\text{only}, \end{array}\right.$$ (5)

where (*x*_*ij*_, *y*_*ij*_, *z*_*ij*_) are three Cartesian coordinates of the relative position between atoms *i* and *j*, i.e., ***r***_*ij*_ = ***r***_*i*_ − ***r***_*j*_ = (*x*_*ij*_, *y*_*ij*_, *z*_*ij*_) in the local frame, and *r*_*ij*_ = |***r***_*ij*_| is its norm. In Eq. (5), the order of the neighbors *j* is sorted in ascending order according to their distance to the atom *i*. ***r***_*ij*_ is transformed from the global relative coordinate $r_{i\;j}^{0}$ through

$$r_{i\;j}=r_{i\;j}^{0}\cdot R_{i},$$ (6)

where

$$R_{i}=\left\{e_{i1},e_{i2},e_{i3}\right\}$$ (7)

is the rotation matrix constructed by

$$e_{i1}=e\left(r_{i,a\left(i\right)}\right),$$ (8)

$$e_{i2}=e\left(r_{i,b\left(i\right)}-\left(r_{i,b\left(i\right)}\cdot e_{i1}\right)e_{i1}\right),$$ (9)

$$e_{i3}=e_{i1}\times e_{i2},$$ (10)

where ***e***(***r***_*ij*_) = ***r***_*ij*_/*r*_*ij*_ denotes the operation of normalizing a vector. *a*(*i*) ∈ *n*(*i*) and *b*(*i*) ∈ *n*(*i*) are the two axis atoms used to define the axes of the local frame of atom *i*, which, in general, are the two closest atoms, independently of their species, together with the center atom *i*.

The limitation of the local frame descriptor is that it is not smooth at the cutoff radius and the exchanging of the order of two nearest neighbors [i.e., the swapping of *a*(*i*) and *b*(*i*)], so its usage is limited. We note that the local frame descriptor is the only non-smooth descriptor among all DP descriptors, and we recommend using other descriptors for the usual system.

##### b. Two-body embedding DeepPot-SE.

The two-body embedding smooth edition of the DP descriptor $D^{\;i}\in R^{M\times M_{<}}$ is usually named DeepPot-SE descriptor.^10^ It is noted that the descriptor is a multi-body representation of the local environment of the atom *i*. We call it “two-body embedding” because the embedding network takes only the distance between atoms *i* and *j* (see below), but it is not implied that the descriptor takes only the pairwise information between *i* and its neighbors. The descriptor, using either full information or radial-only information, is given by

$$D^{\;i}=\left\{\begin{array}{cc} \frac{1}{N_{c}^{2}}{\left(G^{\;i}\right)}^{T}R^{\;i}{\left(R^{\;i}\right)}^{T}G_{<}^{i},\; & \text{full},\\ \frac{1}{N_{c}}\sum_{j}{\left(G^{\;i}\right)}_{jk},\; & \text{radial}-\text{only}, \end{array}\right.$$ (11)

where $R^{\;i}\in R^{N_{c}\times \left\{1,4\right\}}$ is the coordinate matrix, and each row of $R^{\;i}$ can be constructed as

$${\left(R^{\;i}\right)}_{j}=\left\{\;,\begin{array}{cc} \left\{\;,\begin{array}{cccc} s\left(r_{i\;j}\right) & \frac{s\left(r_{i\;j}\right)x_{i\;j}}{r_{i\;j}} & \frac{s\left(r_{i\;j}\right)y_{i\;j}}{r_{i\;j}} & \frac{s\left(r_{i\;j}\right)z_{i\;j}}{r_{i\;j}} \end{array},\;\right\},\; & \text{full},\\ \left\{\;\begin{array}{c} s\left(r_{i\;j}\right) \end{array}\;\right\},\; & \text{radial}-\text{only}, \end{array}\right.$$ (12)

where ***r***_*ij*_ = ***r***_*j*_ − ***r***_*i*_ = (*x*_*ij*_, *y*_*ij*_, *z*_*ij*_) is the relative coordinate and *r*_*ij*_ = ‖***r***_*ij*_‖ is its norm. The switching function *s*(*r*) is defined as

$$s\left(r\right)=\left\{\;,\begin{array}{cc} \frac{1}{r},\; & r<r_{s},\\ \frac{1}{r}\left[x^{3}\left(-6x^{2}+15x-10\right)+1\right],\; & r_{s}\leq r<r_{c},\\ 0,\; & r\geq r_{c}, \end{array}\right.$$ (13)

where $x=\frac{r-r_{s}}{r_{c}-r_{s}}$ switches from 0 at *r*_*s*_ to 1 at the cutoff radius *r*_*c*_ and $\left[x^{3}\left(-6x^{2}+15x-10\right)+1\right]$ switches from 1 at *r*_*s*_ to 0 at *r*_*c*_. The switching function *s*(*r*) is smooth in the sense that the second-order derivative is continuous. The derivation process of the fifth-order polynomial $\left[x^{3}\left(-6x^{2}+15x-10\right)+1\right]$ can be found in Appendix A.

Each row of the embedding matrix $G^{\;i}\in R^{N_{c}\times M}$ consists of *M* nodes from the output layer of an NN function $N_{\;e,2}$ of *s*(*r*_*ij*_),

$${\left(G^{\;i}\right)}_{j}=N_{\;e,2}\left(s\left(r_{i\;j}\right)\right),$$ (14)

where the NN function N was given in Eq. (4) and the subscript “*e*, 2” is used to distinguish the NN from other NNs used in the DP model. In Eq. (14), the network parameters are not explicitly written. $G_{<}^{i}\in R^{N_{c}\times M_{<}}$ only takes first *M*_<_ columns of $G^{\;i}$ to reduce the size of $D^{\;i}$. *r*_*s*_, *r*_*c*_, *M*, and *M*_<_ are hyperparameters provided by the user. Compared to the local frame descriptor, the DeepPot-SE is continuous up to the second-order derivative in its domain.

##### c. Three-body embedding DeepPot-SE.

The three-body embedding DeepPot-SE descriptor incorporates bond-angle information, making the model more accurate.^50^ The descriptor $D^{\;i}$ can be represented as

$$D^{\;i}=\frac{1}{N_{c}^{2}}\left(R^{\;i}{\left(R^{\;i}\right)}^{T}\right):G^{\;i},$$ (15)

where $R^{\;i}$ is defined by Eq. (12). Currently, only the full information case of $R^{\;i}$ is supported by the three-body embedding. Similar to Eq. (14), each element of $G^{\;i}\in R^{N_{c}\times N_{c}\times M}$ comes from *M* nodes from the output layer of an NN $N_{\;e,3}$ function,

$${\left(G^{\;i}\right)}_{jk}=N_{\;e,3}\left({\left(\theta_{i}\right)}_{jk}\right),$$ (16)

where ${\left(\theta_{i}\right)}_{jk}={\left(R^{\;i}\right)}_{j}\cdot {\left(R^{\;i}\right)}_{k}$ considers the angle form of two neighbors (*j* and *k*). The notation “:” in Eq. (15) indicates the contraction between matrix $R^{\;i}{\left(R^{\;i}\right)}^{T}$ and the first two dimensions of tensor $G^{\;i}$. The network parameters are also not explicitly written in Eq. (16).

##### d. Handling the systems composed of multiple chemical species.

For a system with multiple chemical species (|{*α*_*i*_}| > 1), parameters of the embedding network $N_{\;e,\left\{2,3\right\}}$ are as follows chemical-species-wise in Eqs. (14) and (16):

$${\left(G^{\;i}\right)}_{j}=N_{\;e,2}^{\alpha_{i},\alpha_{j}}\left(s\left(r_{i\;j}\right)\right)\;or\;{\left(G^{\;i}\right)}_{j}=N_{\;e,2}^{\alpha_{j}}\left(s\left(r_{i\;j}\right)\right),$$ (17)

$${\left(G^{\;i}\right)}_{jk}=N_{\;e,3}^{\alpha_{j},\alpha_{k}}\left({\left(\theta_{i}\right)}_{jk}\right).$$ (18)

Thus, there will be $N_{t}^{2}$ or *N*_*t*_ embedding networks, where *N*_*t*_ is the number of chemical species. To improve the performance of matrix operations, *n*(*i*) is divided into blocks of different chemical species. Each matrix with a dimension of *N*_*c*_ is divided into corresponding blocks, and each block is padded to $N_{c}^{\alpha_{j}}$ separately. The limitation of this approach is that when there are large numbers of chemical species, such as 57 elements in the OC2M dataset,^115,116^ the number of embedding networks will become 3249 or 57, requiring large memory and decreasing computing efficiency.

##### e. Type embedding.

To reduce the number of NN parameters and improve computing efficiency when there are large numbers of chemical species, the type embedding A is introduced, represented as a NN function $N_{t}$ of the atomic type *α*,

$$A^{\;i}=N_{t}\left(\text{one\_hot}\left(\alpha_{i}\right)\right),$$ (19)

where *α*_*i*_ is converted to a one-hot vector representing the chemical species before feeding to the NN. The NN function N was given in Eq. (4). Based on Eqs. (14) and (16), the type embeddings of central and neighboring atoms $A^{\;i}$ and $A^{\;j}$ are added as an extra input of the embedding network $N_{\;e,\left\{2,3\right\}}$,

$${\left(G^{\;i}\right)}_{j}=N_{\;e,2}\left(\left\{s\left(r_{i\;j}\right),A^{\;i},A^{\;j}\right\}\right)\;or\;{\left(G^{\;i}\right)}_{j}=N_{\;e,2}\left(\left\{s\left(r_{i\;j}\right),A^{\;j}\right\}\right),$$ (20)

$${\left(G^{\;i}\right)}_{jk}=N_{\;e,3}\left(\left\{{\left(\theta_{i}\right)}_{jk},A^{\;j},A^{\;k}\right\}\right).$$ (21)

In this way, all chemical species share the same network parameters through the type embedding.

##### f. Attention-based descriptor.

An attention-based descriptor $D^{\;i}\in R^{M\times M_{<}}$, which is proposed in the pretrainable DPA-1^51^ model, is given by

$$D^{\;i}=\frac{1}{N_{c}^{2}}{\left({\hat{G}}^{\;i}\right)}^{T}R^{\;i}{\left(R^{\;i}\right)}^{T}{\hat{G}}_{<}^{i},$$ (22)

where ${\hat{G}}^{\;i}$ represents the embedding matrix $G^{\;i}$ after additional self-attention mechanism^119^ and $R^{\;i}$ is defined by the full case in Eq. (12). Note that we obtain $G^{\;i}$ from Eq. (20) using the type embedding method by default in this descriptor.

To perform the self-attention mechanism, the queries $Q^{\;i,l}\in R^{N_{c}\times d_{k}}$, keys $K^{\;i,l}\in R^{N_{c}\times d_{k}}$, and values $V^{i,l}\in R^{N_{c}\times d_{v}}$ are first obtained,

$${\left(Q^{\;i,l}\right)}_{\;j}=Q_{l}\left({\left(G^{\;i,l-1}\right)}_{\;j}\right),$$ (23)

$${\left(K^{\;i,l}\right)}_{\;j}=K_{l}\left({\left(G^{\;i,l-1}\right)}_{\;j}\right),$$ (24)

$${\left(V^{i,l}\right)}_{\;j}=V_{l}\left({\left(G^{\;i,l-1}\right)}_{\;j}\right),$$ (25)

where *Q*_*l*_, *K*_*l*_, *V*_*l*_ represent three trainable linear transformations that output the queries and keys of dimension *d*_*k*_ and values of dimension *d*_*v*_ and *l* is the index of the attention layer. The input embedding matrix to the attention layers, denoted by $G^{\;i,0}$, is chosen as the two-body embedding matrix (14).

Then, the scaled dot-product attention method^119,118^ is adopted,

$$A\left(Q^{\;i,l},K^{\;i,l},V^{i,l},R^{\;i,l}\right)=\varphi \left(Q^{\;i,l},K^{\;i,l},R^{\;i,l}\right)V^{i,l},$$ (26)

where $\varphi \left(Q^{\;i,l},K^{\;i,l},R^{\;i,l}\right)\in R^{N_{c}\times N_{c}}$ is attention weights. In the original attention method, one typically has $\varphi \left(Q^{\;i,l},K^{\;i,l}\right)=softmax\left(\frac{Q^{\;i,l}{\left(K^{\;i,l}\right)}^{T}}{\sqrt{d_{k}}}\right)$, with $\sqrt{d_{k}}$ being the normalization temperature. This is slightly modified to incorporate the angular information,

$$\varphi \left(Q^{\;i,l},K^{\;i,l},R^{\;i,l}\right)=softmax\left(\frac{Q^{\;i,l}{\left(K^{\;i,l}\right)}^{T}}{\sqrt{d_{k}}}\right)⊙{\hat{R}}^{\;i}{\left({\hat{R}}^{\;i}\right)}^{T},$$ (27)

where ${\hat{R}}^{\;i}\in R^{N_{c}\times 3}$ denotes normalized relative coordinates, ${\hat{R}}_{\;j}^{i}=\frac{r_{i\;j}}{\left\|r_{i\;j}\right\|}$, and ⊙ means element-wise multiplication.

Then, layer normalization is added in a residual way to finally obtain the self-attention local embedding matrix ${\hat{G}}^{i}=G^{\;i,L_{a}}$ after *L*_*a*_ attention layers,

$$G^{\;i,l}=G^{\;i,l-1}+LayerNorm\left(A\left(Q^{\;i,l},K^{\;i,l},V^{i,l},R^{\;i,l}\right)\right).$$ (28)

##### g. Hybrid descriptor.

A hybrid descriptor $D_{\text{hyb}}^{i}$ concatenates multiple kinds of descriptors into one descriptor,^53^

$$D_{\text{hyb}}^{i}=\left\{\;\begin{array}{cccc} D_{1}^{i} & D_{2}^{i} & \cdots & D_{n}^{i} \end{array}\;\right\}.$$ (29)

The list of descriptors can be different types or the same descriptors with different parameters. This way, one can set the different cutoff radii for different descriptors.

##### h. Compression.

The compression of the DP model uses three techniques, tabulated inference, operator merging, and precise neighbor indexing, to improve the performance of model training and inference when the model parameters are properly trained.^109^

For better performance, the NN inference can be replaced by tabulated function evaluations if the input of the NN is of dimension one. The embedding networks $N_{\;e,2}$ defined by (14) and $N_{\;e,3}$ defined by (16) are of this type. The idea is to approximate the output of the NN by a piece-wise polynomial fitting. The input domain (a compact domain in R) is divided into *L*_*c*_ equally spaced intervals, in which we apply a fifth-order polynomial $g_{m}^{l}\left(x\right)$ approximation of the *m*th output component of the NN function,

$$g_{m}^{l}\left(x\right)=a_{m}^{l}x^{5}+b_{m}^{l}x^{4}+c_{m}^{l}x^{3}+d_{m}^{l}x^{2}+e_{m}^{l}x+f_{\;m}^{l},\;x\in \left[x_{l},x_{l+1}\right),$$ (30)

where *l* = 1, 2, …, *L*_*c*_ is the index of the intervals, $x_{1},\ldots ,x_{L_{c}},x_{L_{c}+1}$ are the endpoints of the intervals, and $a_{m}^{l}$, $b_{m}^{l}$, $c_{m}^{l}$, $d_{m}^{l}$, $e_{m}^{l}$, and $f_{\;m}^{l}$ are the fitting parameters. The fitting parameters can be computed by using the following equations:

$$\begin{array}{ll} a_{m}^{l} & =\frac{1}{2\Delta x_{l}^{5}}\left[12h_{m,l}-6\left(y_{m,l+1}^{\prime }+y_{m,l}^{\prime }\right)\Delta x_{l}\right.\\ & \left.\;+\left(y_{m,l+1}^{\prime \prime }-y_{m,l}^{\prime \prime }\right)\Delta x_{l}^{2}\right], \end{array}$$ (31)

$$\begin{array}{ll} b_{m}^{l} & =\frac{1}{2\Delta x_{l}^{4}}\left[-30h_{m,l}+\left(14y_{m,l+1}^{\prime }+16y_{m,l}^{\prime }\right)\Delta x_{l}\right.\\ & \left.\;+\left(-2y_{m,l+1}^{\prime \prime }+3y_{m,l}^{\prime \prime }\right)\Delta x_{l}^{2}\right], \end{array}$$ (32)

$$\begin{array}{ll} c_{m}^{l} & =\frac{1}{2\Delta x_{l}^{3}}\left[20h_{m,l}-\left(8y_{m,l+1}^{\prime }+12y_{m,l}^{\prime }\right)\Delta x_{l}\right.\\ & \left.\;+\left(y_{m,l+1}^{\prime \prime }-3y_{m,l}^{\prime \prime }\right)\Delta x_{l}^{2}\right], \end{array}$$ (33)

$$d_{m}^{l}=\frac{1}{2}y_{m,l}^{\prime \prime },$$ (34)

$$e_{m}^{l}=y_{m,l}^{\prime },$$ (35)

$$f_{\;m}^{l}=y_{m,l},$$ (36)

where Δ*x*_*l*_ = *x*_*l*+1_ − *x*_*l*_ denotes the size of the interval. *h*_*m*,*l*_ = *y*_*m*,*l*+1_ − *y*_*m*,*l*_. *y*_*m*,*l*_ = *y*_*m*_(*x*_*l*_), $y_{m,l}^{\prime }=y_{m}^{\prime }\left(x_{l}\right)$, and $y_{m,l}^{\prime \prime }=y_{m}^{\prime \prime }\left(x_{l}\right)$ are the value, the first-order derivative, and the second-order derivative of the *m*th component of the target NN function at the interval point *x*_*l*_, respectively. The first- and second-order derivatives are easily calculated by the back-propagation of the NN functions.

In the standard DP model inference, taking the two-body embedding descriptor as an example, the matrix product ${\left(G^{\;i}\right)}^{T}R$ requires the transfer of the tensor $G^{\;i}$ between the register and the host/device memories, which usually becomes the bottle-neck of the computation due to the relatively small memory bandwidth of the GPUs. The compressed DP model merges the matrix multiplication ${\left(G^{\;i}\right)}^{T}R$ with the tabulated inference step. More specifically, once one column of ${\left(G^{\;i}\right)}^{T}$ is evaluated, it is immediately multiplied with one row of the environment matrix in the register, and the outer product is deposited to the result of ${\left(G^{\;i}\right)}^{T}R$. By the operator merging technique, the allocation of $G^{\;i}$ and the memory movement between register and host/device memories is avoided. The operator merging of the three-body embedding can be derived analogously.

The first dimension, *N*_*c*_, of the environment $\left(R^{\;i}\right)$ and embedding $\left(G^{\;i}\right)$ matrices is the expected maximum number of neighbors. If the number of neighbors of an atom is smaller than *N*_*c*_, the corresponding positions of the matrices are pad with zeros. In practice, if the real number of neighbors is significantly smaller than *N*_*c*_, a notable operation is spent on the multiplication of padding zeros. In the compressed DP model, the number of neighbors is precisely indexed at the tabulated inference stage, further saving computational costs.

#### 3. Fitting networks

The fitting network can fit the potential energy of a system, along with the force and the virial, and tensorial properties, such as the dipole and the polarizability.

##### a. Fitting potential energies.

In the DP model (1), we let the fitting network $F_{0}$ map the descriptor $D^{\;i}$ to a scalar, where the subscript “0” means that the output is a zero-order tensor (i.e., scalar). The model can then be used to predict the total potential energy of the system by

$$E=\underset{i}{\sum }E_{i}=\underset{i}{\sum }F_{0}\left(D^{\;i}\right),$$ (37)

where the output of the fitting network is treated as the atomic potential energy contribution, i.e., *E*_*i*_. The output scalar can also be treated as other scalar properties defined on an atom, for example, the partial charge of atom *i*.

In some cases, atomic-specific or frame-specific parameters, such as electron temperature,^119^ may be treated as extra input to the fitting network. We denote the atomic and frame-specific parameters by $P^{i}\in R^{N_{p}}$ (with *N*_*p*_ being the dimension) and $Q\in R^{N_{q}}$ (with *N*_*q*_ being the dimension), respectively,

$$E_{i}=F_{0}\left(\left\{D^{\;i},P^{i},Q\right\}\right).$$ (38)

The atomic force ***F***_*i*_ and the virial tensor **Ξ** = (Ξ_*αβ*_) (if PBC is applied) can be derived from the potential energy *E*,

$$F_{i,\alpha }=-\frac{\partial E}{\partial r_{i,\alpha }},$$ (39)

$$\Xi_{\alpha \beta }=-\underset{\gamma }{\sum }\frac{\partial E}{\partial h_{\gamma \alpha }}h_{\gamma \beta },$$ (40)

where *r*_*i*,*α*_ and *F*_*i*,*α*_ denote the *α*th component of the coordinate and force of atom *i*. *h*_*αβ*_ is the *β*th component of the *α*th basis vector of the simulation region.

##### b. Fitting tensorial properties.

To represent the first-order tensorial properties (i.e., vector properties), we let the fitting network, denoted by $F_{1}$, output an *M*-dimensional vector; then, we have the representation

$${\left(T_{i}^{\left(1\right)}\right)}_{\alpha }=\frac{1}{N_{c}}\overset{N_{c}}{\underset{j=1}{\sum }}\overset{M}{\underset{m=1}{\sum }}{\left(G^{\;i}\right)}_{jm}{\left(R^{\;i}\right)}_{j,\alpha +1}{\left(F_{1}\left(D^{\;i}\right)\right)}_{m},\;\alpha =1,2,3.$$ (41)

We let the fitting network $F_{2}$ output an *M*-dimensional vector, and the second-order tensorial properties (matrix properties) are formulated as

$$\begin{array}{cc} {\left(T_{i}^{\left(2\right)}\right)}_{\alpha \beta } & =\frac{1}{N_{c}^{2}}\overset{N_{c}}{\underset{j=1}{\sum }}\overset{N_{c}}{\underset{k=1}{\sum }}\overset{M}{\underset{m=1}{\sum }}{\left(G^{\;i}\right)}_{jm}{\left(R^{\;i}\right)}_{j,\alpha +1}{\left(R^{\;i}\right)}_{k,\beta +1}\\ & \;\times {\left(G^{\;i}\right)}_{km}{\left(F_{2}\left(D^{\;i}\right)\right)}_{m},\\ & \;\;\;\alpha ,\beta =1,2,3, \end{array}$$ (42)

where $G^{\;i}$ and $R^{\;i}$ can be found at Eqs. (12) and (14) (full case), respectively. Thus, the tensor fitting network requires the descriptor to have the same or similar form as the DeepPot-SE descriptor. The NN functions $F_{1}$ and $F_{2}$ were given in Eq. (4). The total tensor ***T*** (total dipole ***T***^(1)^ or total polarizability ***T***^(2)^) is the sum of the atomic tensor,

$$T=\underset{i}{\sum }T_{i}.$$ (43)

The tensorial models can be used to calculate the IR spectrum^105^ and Raman spectrum.^106^

##### c. Handling the systems composed of multiple chemical species.

Similar to the embedding networks, if the type embedding approach is not used, the fitting network parameters are chemical-species-wise, and there are *N*_*t*_ sets of fitting network parameters. For performance, atoms are sorted by their chemical species *α*_*i*_ in advance. Taking an example, the atomic energy *E*_*i*_ is represented as follows based on Eq. (38):

$$E_{i}=F_{\;0}^{{\;\alpha }_{i}}\left(D^{\;i}\right).$$ (44)

When the type embedding is used, all chemical species share the same network parameters, and the type embedding is inserted into the input of the fitting networks in Eq. (38),

$$E_{i}=F_{0}\left(\left\{D^{\;i},A^{\;i}\right\}\right).$$ (45)

#### 4. Deep potential range correction (DPRc)

Deep Potential-Range Correction (DPRc)^52,75^ was initially designed to correct the potential energy from a fast, linear-scaling low-level semiempirical QM/MM theory to a high-level *ab initio* QM/MM theory in a range-correction way to quantitatively correct short and mid-range non-bonded interactions leveraging the non-bonded lists routinely used in molecular dynamics simulations using molecular mechanical force fields, such as AMBER.^120^ In this way, long-ranged electrostatic interactions can be modeled efficiently using the particle mesh Ewald method^120^ or its extensions for multipolar^94,95^ and QM/MM^96,97^ potentials. In a DPRc model, the switch function in Eq. (13) is modified to disable MM–MM interaction,

$$s_{\text{DPRc}}\left(r_{i\;j}\right)=\left\{\begin{array}{cc} 0\; & \text{if }i\;\in \text{MM}\wedge j\in \text{MM},\\ s\left(r_{i\;j}\right)\; & \text{otherwise}, \end{array}\right.$$ (46)

where *s*_DPRc_(*r*_*ij*_) is the new switch function and *s*(*r*_*ij*_) is the old one in Eq. (13). This ensures that the forces between MM atoms are zero, i.e.,

$$F_{i\;j}=-\frac{\partial E}{\partial r_{i\;j}}=0,\;i\;\in \text{MM}\wedge j\;\in \text{MM}.$$ (47)

The fitting network in Eq. (38) is revised to remove energy bias from MM atoms,

$$E_{i}=\left\{\;,\begin{array}{cc} F_{0}\left(D^{\;i}\right)\; & \text{if }i\in \text{QM},\\ F_{0}\left(D^{\;i}\right)-F_{0}\left(0\right)\; & \text{if }i\in \text{MM}, \end{array}\right.$$ (48)

where **0** is a zero matrix. It is worth mentioning that the usage of DPRc is not limited to its initial design for QM/MM correction and can be expanded to any similar interaction.^121^

#### 5. Deep potential long range (DPLR)

The Deep Potential Long Range (DPLR) model adds the electrostatic energy to the total energy,^53^

$$E=E_{\text{DP}}+E_{\text{ele}},$$ (49)

where *E*_DP_ is the short-range contribution constructed as the standard energy model in Eq. (37) that is fitted against (E* − *E*_ele_). *E*_ele_ is the electrostatic energy introduced by a group of Gaussian distributions that is an approximation of the electronic structure of the system and is calculated in Fourier space by

$$E_{\text{ele}}=\frac{1}{2\pi V}\underset{m\neq 0,\|m\|\leq L}{\sum }\frac{\exp\left(-\pi^{2}m^{2}/\beta^{2}\right)}{m^{2}}S^{2}\left(m\right),$$ (50)

where *β* is a freely tunable parameter that controls the spread of the Gaussians. *L* is the cutoff in Fourier space, and *S*(*m*), the structure factor, is given by

$$S\left(m\right)=\underset{i}{\sum }q_{i}e^{-2\pi ımr_{i}}+\underset{n}{\sum }q_{n}e^{-2\pi ımW_{n}}\;,$$ (51)

where $ı=\sqrt{-1}$ denotes the imaginary unit, ***r***_*i*_ indicates ion coordinates, *q*_*i*_ is the charge of the ion *i*, and *W*_*n*_ is the *n*th Wannier centroid (WC), which can be obtained from a separated dipole model in Eq. (42). It can be proved that the error in the electrostatic energy introduced by the Gaussian approximations is dominated by a summation of dipole-quadrupole interactions that decay as *r*^−4^, where *r* is the distance between the dipole and quadrupole.^53^

#### 6. Interpolation with a pairwise potential

In applications such as the radiation damage simulation, the interatomic distance may become too close so that the DFT calculations fail. In such cases, the DP model that is an approximation of the DFT potential energy surface is usually replaced by an empirical potential, such as the Ziegler–Biersack–Littmark (ZBL)^122^ screened nuclear repulsion potential in radiation damage simulations.^123^ The DeePMD-kit package supports the interpolation between DP and an empirical pairwise potential,

$$E_{i}=\left(1-w_{i}\right)E_{i}^{\text{DP}}+w_{i}E_{i}^{\text{pair}},$$ (52)

where *w*_*i*_ is the interpolation weight and $E_{i}^{\text{pair}}$ is the atomic contribution due to the pairwise potential *u*^pair^(*r*), i.e.,

$$E_{i}^{\text{pair}}=\underset{j\in n\left(i\right)}{\sum }u^{\text{pair}}\left(r_{i\;j}\right).$$ (53)

The interpolation weight *w*_*i*_ is defined by

$$w_{i}=\left\{\begin{array}{cc} 1,\; & \sigma_{i}<r_{a},\\ u_{i}^{3}\left(-6u_{i}^{2}+15u_{i}-10\right)+1,\; & r_{a}\leq \sigma_{i}<r_{b},\\ 0,\; & \sigma_{i}\geq r_{b}, \end{array}\right.$$ (54)

where *u*_*i*_ = (*σ*_*i*_ − *r*_*a*_)/(*r*_*b*_ − *r*_*a*_). The derivation process of Eq. (54) can be found in Appendix A. In the range [*r*_*a*_, *r*_*b*_], the DP model smoothly switched off and the pairwise potential smoothly switched on from *r*_*b*_ to *r*_*a*_. *σ*_*i*_ is the softmin of the distance between atom *i* and its neighbors,

$$\sigma_{i}=\frac{\sum_{j\in n\left(i\right)}r_{i\;j}e^{-r_{i\;j}/\alpha_{s}}}{\sum_{j\in n\left(i\right)}e^{-r_{i\;j}/\alpha_{s}}},$$ (55)

where the scale *α*_*s*_ is a tunable scale of the interatomic distance *r*_*ij*_. The pairwise potential *u*^pair^(*r*) is defined by a user-defined table that provides the value of *u*^pair^ on an evenly discretized grid from 0 to the cutoff distance.

### B. Trainer

Based on DP models M defined in Eq. (1), a trainer should also be defined to train parameters in the model, including weights and biases in Eq. (4). The learning rate *γ*, the loss function *L*, and the training process should be given in a trainer.

#### 1. Learning rate

The learning rate *γ* decays exponentially,

$$\gamma \left(\tau \right)=\gamma^{0}r^{\left\lfloor \tau /s\right\rfloor },$$ (56)

where τ∈N is the index of the training step, $\gamma^{0}\in R$ is the learning rate at the first step, and the decay rate *r* is given by

$$r={\left(\frac{\gamma^{\text{stop}}}{\gamma^{0}}\right)}^{\frac{s}{\tau^{\text{stop}}}},$$ (57)

where $\tau^{\text{stop}}\in N$, $\gamma^{\text{stop}}\in R$, and s∈N are the stopping step, the stopping learning rate, and the decay steps, respectively, all of which are hyperparameters provided in advance.

#### 2. Loss function

The loss function *L* is given by a weighted sum of different fitting property loss *L*_*p*_,

$$L\left(x;\theta ,\tau \right)=\frac{1}{B}\underset{k\in B}{\sum }\underset{\eta }{\sum }p_{\eta }\left(\tau \right)L_{\eta }\left(x^{k};\theta \right),$$ (58)

where B is the mini-batch of data. ***x*** = {***x***^*k*^} is the dataset. $x^{k}=\left(x_{1}^{k},\ldots ,x_{N}^{k}\right)$ is a single data frame from the set and is composed of all the degrees of freedom of the atoms. *η* denotes the property to be fit. For each property, *p*_*η*_ is a prefactor given by

$$p_{\eta }\left(\tau \right)=p_{\eta }^{\text{limit}}\left(1-\frac{\gamma \left(\tau \right)}{\gamma^{0}}\right)+p_{\eta }^{\text{start}}\frac{\gamma \left(\tau \right)}{\gamma^{0}},$$ (59)

where $p_{\eta }^{\text{start}}$ and $p_{\eta }^{\text{limit}}$ are hyperparameters that give the prefactor at the first training step and the infinite training steps, respectively. *γ*(*τ*) is the learning rate defined by Eq. (56).

The loss function of a specific fitting property *L*_*η*_ is defined by the mean squared error (MSE) of a data frame and is normalized by the number of atoms *N* if *η* is a frame property that is a linear combination of atomic properties. Taking an example, if an energy model is fitted as given in Eq. (37), the properties *η* could be energy *E*, force ***F***, virial **Ξ**, relative energy Δ*E*,^72^ or any combination among them, and the loss functions of them are

$$L_{E}\left(x;\theta \right)=\frac{1}{N}{\left(E\left(x;\theta \right)-E^{*}\right)}^{2},$$ (60)

$$L_{F}\left(x;\theta \right)=\frac{1}{3N}\overset{N}{\underset{k=1}{\sum }}\overset{3}{\underset{\alpha =1}{\sum }}{\left(F_{k,\alpha }\left(x;\theta \right)-F_{k,\alpha }^{*}\right)}^{2},$$ (61)

$$L_{\Xi }\left(x;\theta \right)=\frac{1}{9N}\overset{3}{\underset{\alpha ,\beta =1}{\sum }}{\left(\Xi_{\alpha \beta }\left(x;\theta \right)-\Xi_{\alpha \beta }^{*}\right)}^{2},$$ (62)

$$L_{\Delta E}\left(x;\theta \right)=\frac{1}{N}{\left(\Delta E\left(x;\theta \right)-{\Delta E}^{*}\right)}^{2},$$ (63)

where *F*_*k*,*α*_ is the *α*th component of the force on atom *k* and the superscript “*” indicates the label of the property that should be provided in advance. Using *N* ensures that each loss of fitting property is averaged over atomic contributions before they contribute to the total loss by weight.

If part of atoms is more important than others, for example, certain atoms play an essential role when calculating free energy profiles or kinetic isotope effects,^52,75^ the MSE of atomic forces with prefactors *q*_*k*_ can also be used as the loss function,

$$L_{F}^{p}\left(x;\theta \right)=\frac{1}{3N}\overset{N}{\underset{k=1}{\sum }}\underset{\alpha }{\sum }q_{k}{\left(F_{k,\alpha }\left(x;\theta \right)-F_{k,\alpha }^{*}\right)}^{2}.$$ (64)

The atomic forces with larger prefactors will be fitted more accurately than those in other atoms.

If some forces are quite large, for example, forces can be greater than 60 eV/Å in high-temperature reactive simulations,^77,78^ one may also prefer that the force loss is relative to the magnitude instead of Eq. (61),

$$L_{F}^{r}\left(x;\theta \right)=\frac{1}{3N}\overset{N}{\underset{k=1}{\sum }}\underset{\alpha }{\sum }{\left(\frac{F_{k,\alpha }\left(x;\theta \right)-F_{k,\alpha }^{*}}{|F_{k}^{*}|+\nu }\right)}^{2},$$ (65)

where *ν* is a small constant used to protect an atom where the magnitude of $F_{k}^{*}$ is small from having a large $L_{F}^{r}$. Benefiting from the relative force loss, small forces can be fitted more accurately.

#### 3. Training process

During the training process, the loss function is minimized by the stochastic gradient descent algorithm Adam.^124^ Ideally, the resulting parameter is the minimizer of the loss function,

$$\theta^{*}=\underset{\theta }{\mathrm{arg min}}\underset{\tau \to +\infty }{\lim}L\left(x;\theta ,\tau \right).$$ (66)

In practice, the Adam optimizer stops at the step *τ*_stop_, and the learning rate varies according to scheme (56). *τ*_stop_ is a hyperparameter usually set to several million.

#### 4. Multiple task training

The multi-task training process can simultaneously handle different datasets with properties that cannot be fitted in one network (e.g., properties from DFT calculations under different exchange–correlation functionals or different basis sets). These datasets are denoted by $x^{\left(1\right)},\ldots ,x^{\left(n_{t}\right)}$. For each dataset, a training task is defined as

$$\underset{\theta }{\min}L^{\left(t\right)}\left(x^{\left(t\right)};\theta^{\left(t\right)},\tau \right),\;t=1,\ldots ,n_{t}.$$ (67)

During the multi-task training process, all tasks share one descriptor with trainable parameters ***θ***_*d*_, while each of them has its own fitting network with trainable parameters $\theta_{f}^{\left(t\right)}$; thus, $\theta^{\left(t\right)}=\left\{\theta_{d},\theta_{f}^{\left(t\right)}\right\}$. At each training step, a task is randomly picked from 1, …, *n*_*t*_, and the Adam optimizer is executed to minimize *L*^(*t*)^ for one step to update the parameter ***θ***^(*t*)^. If different fitting networks have the same architecture, they can share the parameters of some layers to improve training efficiency.

### C. Model deviation

Model deviation *ϵ*_*y*_ is the standard deviation of properties ***y*** inferred by an ensemble of models $M_{1},\ldots ,M_{n_{m}}$ that are trained by the same dataset(s) with the model parameters initialized independently. The DeePMD-kit supports ***y*** to be the atomic force ***F***_*i*_ and the virial tensor **Ξ**. The model deviation is used to estimate the error of a model at a certain data frame, denoted by ***x***, containing the coordinates and chemical species of all atoms. We present the model deviation of the atomic force and the virial tensor,

$$\epsilon_{F,i}\left(x\right)=\sqrt{〈\|F_{i}\left(x;\theta_{k}\right)-〈F_{i}\left(x;\theta_{k}\right)〉{\|}^{2}〉},$$ (68)

$$\epsilon_{\Xi ,\alpha \beta }\left(x\right)=\frac{1}{N}\sqrt{〈{\left(\Xi_{\alpha \beta }\left(x;\theta_{k}\right)-〈\Xi_{\alpha \beta }\left(x;\theta_{k}\right)〉\right)}^{2}〉},$$ (69)

where ***θ***_*k*_ is the parameter of the model $M_{k}$ and the ensemble average ⟨·⟩ is estimated by

$$\left\langle y\left(x;\theta_{k}\right)\right\rangle =\frac{1}{n_{m}}\overset{n_{m}}{\underset{k=1}{\sum }}y\left(x;\theta_{k}\right).$$ (70)

Small *ϵ*_***F***,*i*_ means the model has learned the given data; otherwise, it is not covered, and the training data needs to be expanded. If the magnitude of ***F***_*i*_ or **Ξ** is quite large, a relative model deviation *ϵ*_***F***,*i*,rel_ or *ϵ*_**Ξ**,*αβ*,rel_ can be used instead of the absolute model deviation,^78^

$$\epsilon_{F,i,\text{rel}}\left(x\right)=\frac{\left|\epsilon_{F,i}\left(x\right)\right|}{|〈F_{i}\left(x;\theta_{k}\right)〉|+\nu },$$ (71)

$$\epsilon_{\Xi ,\alpha \beta ,\text{rel}}\left(x\right)=\frac{\epsilon_{\Xi ,\alpha \beta }\left(x\right)}{|〈\Xi \left(x;\theta_{k}\right)〉|+\nu },$$ (72)

where *ν* is a small constant used to protect an atom where the magnitude of ***F***_*i*_ or **Ξ** is small from having a large model deviation.

Statistics of *ϵ*_***F***,*i*_ and *ϵ*_**Ξ**,*αβ*_ can be provided, including the maximum, average, and minimal model deviation over the atom index *i* and over the component index *α*, *β*, respectively. The maximum model deviation of forces *ϵ*_***F***,max_ in a frame was found to be the best error indicator in a concurrent or active learning algorithm.^103,107^

## III. TECHNICAL IMPLEMENTATION

In addition to incorporating new powerful features, DeePMD-kit has been designed with the following goals in mind: high performance, high usability, high extensibility, and community engagement. These goals are crucial for DeePMD-kit to become a widely-used platform across various computational fields. In this section, we will introduce several technical implementations that have been put in place to achieve these goals.

### A. Code architecture

**FIG. 2.**
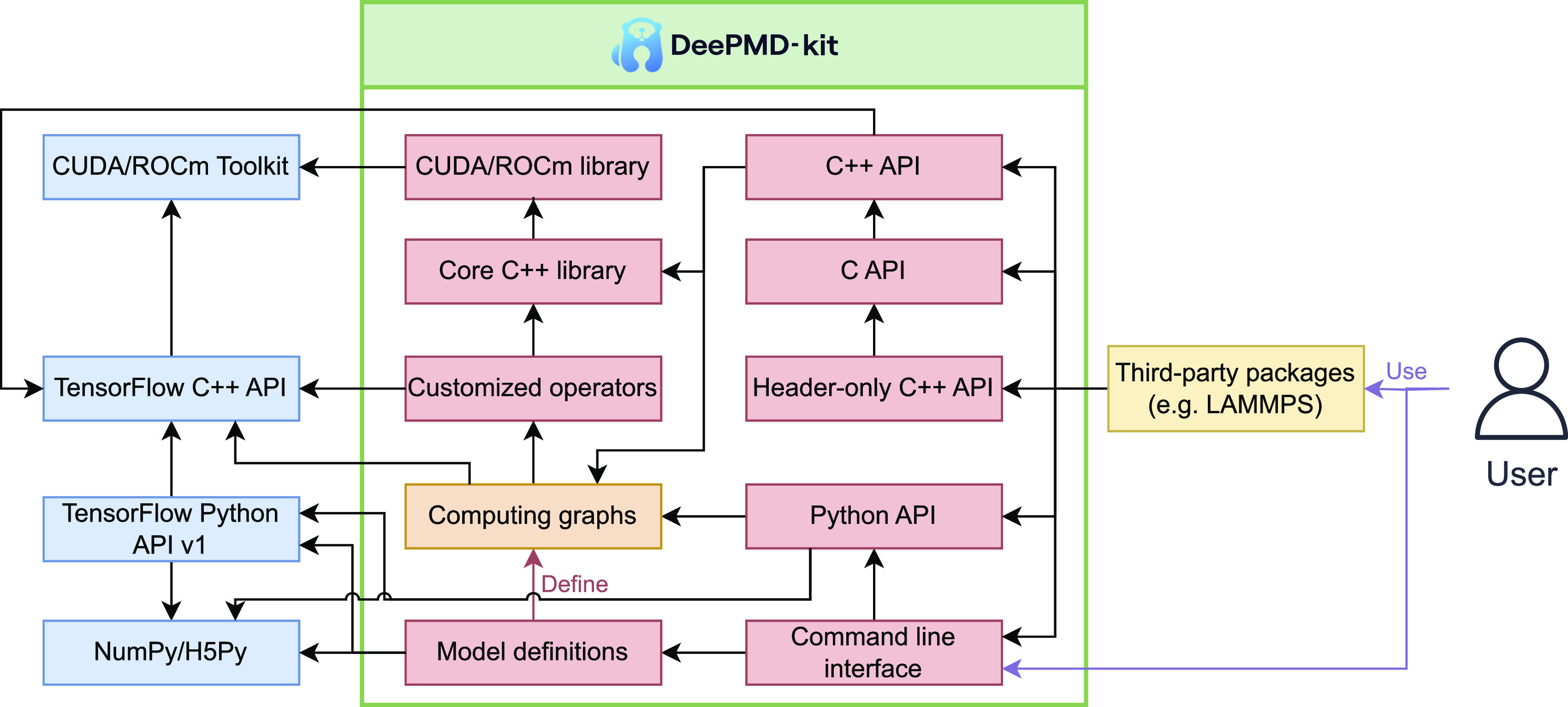
The architecture of the DeePMD-kit code. The red boxes are modules within the DeePMD-kit package (the green box), the orange box represents computing graphs, the blue boxes are dependencies of the DeePMD-kit, and the yellow box represents third-party packages integrated with DeePMD-kit, including LAMMPS, i-PI, GROMACS, AMBER, OpenMM, ABACUS, ASE, MAGUS, DP-Data, DP-GEN, and MLatom. Customized operators are operators that are not offered by TensorFlow, including atomic environmental matrix, interpolation with a pairwise potential, and tabulated inference of the embedding matrix. The direction of the black arrow A → B indicates that module A is dependent on module B. The red and purple arrows represent “define” and “use,” respectively.

The DeePMD-kit utilizes TensorFlow’s computational graph architecture to construct its DP models,^125^ which are composed of various operators implemented with C++, including customized ones, such as the environment matrix, Ewald summation, compressed operator, and their backward propagations. The auto-grad mechanism provided by TensorFlow is used to compute the derivatives of the DP model with respect to the input atomic coordinates and simulation cell tensors. To optimize performance, some of the critical customized operators are implemented for GPU execution using CUDA or ROCm toolkit libraries. The DeePMD-kit provides Python, C++, and C APIs for inference, facilitating easy integration with third-party software packages. As indicated in Fig. 2, the code of the DeePMD-kit consists of the following modules:

• The core C++ library provides the implementation of customized operators, such as the atomic environmental matrix, neighbor lists, and compressed neural networks. It is important to note that the core C++ library is independently built and tested without TensorFlow’s C++ interface.
• The GPU library (CUDA^126^ or ROCm^127^), an optional part of the core C++ library, is used to compute customized operators on GPU devices other than central processing units (CPUs). This library depends on the GPU toolkit library (NVIDIA CUDA Toolkit or AMD ROCm Toolkit) and is also independently built and tested.
• The DP operators library contains several customized operators not supported by TensorFlow.^125^ TensorFlow provides both Python and C++ interfaces to implement some customized operators, with the TensorFlow C++ library packaged inside its Python package.
• The “model definition” module, written in Python, is used to generate computing graphs composed of TensorFlow operators, DP customized operators, and model parameters organized as “variables.” The graph can be saved into a file that can be restored for inference. It depends on the TensorFlow Python API (version 1, tf.compat.v1) and other Python dependencies, such as the NumPy^128^ and H5Py^129^ packages.
• The Python application programming interface (API) is used for inference and can read computing graphs from a file and use the TensorFlow Python API to execute the graph.
• The C++ API, built on the TensorFlow C++ interface, does the same thing as the Python API for inference.
• The C API is a wrapper of the C++ API and provides the same features as the C++ API. Compared to the C++ API, the C API has a more stable application binary interface (ABI) and ensures backward compatibility.
• The header-only C++ API is a wrapper of the C API and provides the same interface as the C++ API. It has the same stable ABI as the C API but still takes advantage of the flexibility of C++.
• The command line interface (CLI) is provided to both general users and developers and is used for both training and inference. It depends on the model definition module and the Python API.

The CMake build system^130^ manages all modules, and the pip and scikit-build^131^ packages are used to distribute DeePMD-kit as a Python package. The standard Python unit testing framework^132^ is used for unit tests on all Python codes, while GoogleTest software^133^ is used for tests on all C++ codes. GitHub Actions automates build, test, and deployment pipelines.

### B. Performance

#### 1. Hardware acceleration

In the TensorFlow framework, a static graph combines multiple operators with inputs and outputs. Two kinds of operators are time-consuming during training or inference. The first one is TensorFlow’s native operators for neural networks (see Sec. II A 1) and matrix operations, which have been fully optimized by the TensorFlow framework itself^125^ for both CPU and GPU architectures. Second, the DeePMD-kit’s customized operators are for computing the atomic environment [Eqs. (6) and (12)], for interpolation with a pairwise potential, and for the tabulated inference of the embedding matrix [Eq. (30)]. These operators are not supported by the TensorFlow framework but can be accelerated using OpenMP,^134^ CUDA,^126^ and ROCm^127^ for parallelization under both CPUs and GPUs, except the features without GPU supports listed in Appendix B.

The operator of the environment matrix includes two steps:^108^ formatting the neighbor list and computing the matrix elements of R. In the formatting step, the neighbors of the atom *i* are sorted according to their type *α*_*j*_, their distance *r*_*ij*_ to atom *i*, and finally their index *j*. To improve sorting performance on GPUs, the atomic type, distance, and index are compressed into a 64-bit integer S∈N used for sorting,

$$S=\alpha_{j}\times 10^{15}+\left\lfloor r_{i\;j}\times 10^{8}\right\rfloor \times 10^{5}+j.$$ (73)

The sorted neighbor index is decompressed from the sorted *S* and then used to format the neighbor list.

#### 2. MPI implementation for multi-device training and MD simulations

Users may prefer to utilize multiple CPU cores, GPUs, or hardware across multiple nodes to achieve faster performance and larger memory during training or molecular dynamics (MD) simulations. To facilitate this, DeePMD-kit has added message-passing interface (MPI) implementation^135,136^ for multi-device training and MD simulations in two ways, which are described below.

Multi-device training is conducted with the help of Horovod, a distributed training framework.^137^ Horovod works in the data-parallel mode by equally distributing a batch of data among workers along the axis of the batch size B.^138^ During training, each worker consumes sliced input records at different offsets, and only the trainable parameter gradients are averaged with peers. This design avoids batch size and tensor shape conflicts and reduces the number of bytes that need to be communicated among processes. The mpi4py package^139^ is used to remove redundant logs.

Multi-device MD simulations are implemented by utilizing the existing parallelism features of third-party MD packages. For example, a Large-scale Atomic/Molecular Massively Parallel Simulator (LAMMPS) enables parallelism across CPUs by optimizing partitioning, communication, and neighbor lists.^140^ AMBER builds a similar neighbor list in the interface to DeePMD-kit.^52,54,141^ DeePMD-kit supports local atomic environment calculation and accepts the neighbor list *n*(*i*) from other software to replace the native neighbor list calculation.^108^ In a device, the neighbors from other devices are considered “ghost” atoms that do not contribute atomic energy *E*_*i*_ to this device’s total potential energy *E*.

#### 3. Non-von Neumann molecular dynamics (NVNMD)

When performing molecular dynamics (MD) simulations on CPUs and GPUs, a large majority of time and energy (e.g., more than 95%) is consumed by the DP model inference. This inference process is limited by the “memory wall” and “power wall” bottlenecks of von Neumann (vN) architecture, which means that a significant amount of time and energy (e.g., over 90%) is wasted on data transfer between the processor and memory. As a result, it is difficult to improve computational efficiency.

To address these challenges, non-von Neumann molecular dynamics (NVNMD) uses a non-von Neumann (NvN) architecture chip to accelerate inference. The NvN chip contains processing and memory units that can be used to implement the DP algorithm. In the NvN chip, the hardware algorithm runs fully pipelined. The model parameters are stored in on-chip memory after being loaded from off-chip memory during the initialization process. Therefore, two components of data shuttling are avoided: (1) reading/writing the intermediate results from/to off-chip memory and (2) loading model parameters from off-chip memory during the calculation process. As a result, the DP model ensures high accuracy with NVNMD, while the NvN chip ensures high computational efficiency. For more details, see Ref. 110.

### C. Usability

#### 1. Documentation

DeePMD-kit’s features and arguments have grown rapidly with more and more development. To address this issue, we have introduced Sphinx^142^ and Doxygen^143^ to manage and generate documentation for developers from docstrings in the code. We use the DArgs package (see Sec. III E) to automatically generate Sphinx documentation for user input arguments. The documentation is currently hosted on Read the Docs (https://docs.deepmodeling.org/projects/deepmd/). Furthermore, we strive to make the error messages raised by DeePMD-kit clear to users. In addition, the GitHub Discussion forum allows users to ask questions and receive answers. Recently, several tutorials have been published^49,54^ to help new users quickly learn DeePMD-kit.

#### 2. Easy installation

As shown in Fig. 2, DeePMD-kit has dependencies on both Python and C++ libraries of TensorFlow, which can make it difficult and time-consuming for new users to build TensorFlow and DeePMD-kit from the source code. Therefore, we provide compiled binary packages that are distributed via pip, Conda (DeepModeling and conda-forge^144^ channels), Docker, and offline packages for Linux, macOS, and Windows platforms. With the help of these pre-compiled binary packages, users can install DeePMD-kit in just a few minutes. These binary packages include DeePMD-kit’s LAMMPS plugin, i-PI driver, and GROMACS patch. As LAMMPS provides a plugin mode in its latest version, DeePMD-kit’s LAMMPS plugin can be compiled without having to re-compile LAMMPS.^140^ We offer a compiled binary package that includes the C API and the header-only C++ API, making it simpler to integrate with sophisticated software, such as AMBER.^52,54,141^

#### 3. User interface

DeePMD-kit offers a command line interface (CLI) for training, freezing, and testing models. In addition to CLI arguments, users must provide a JSON^145^ or YAML^146^ file with completed arguments for components listed in Sec. II. The DArgs package (see Sec. III E) parses these arguments to check if user input is correct. An example of how to use the user interface is provided in Ref. 54. Users can also use DP-GUI (see Sec. III E) to fill in arguments in an interactive web page and save them to a JSON^145^ file.

DeePMD-kit provides an automatic algorithm that assists new users in deciding on several arguments. For example, the automatic batch size B determines the maximum batch size during training or inferring to fully utilize memory on a GPU card. The automatic neighbor size *N*_*c*_ determines the maximum number of neighbors by stating the training data to reduce model memory usage. The automatic probability determines the probability of using a system during training. These automatic arguments reduce the difficulty of learning and using the DeePMD-kit.

#### 4. Input data

To train and test models, users are required to provide fitting data in a specified format. DeePMD-kit supports two file formats for data input: NumPy binary files^128^ and HDF5 files.^147^ These formats are designed to offer superior performance when read by the program with parallel algorithms compared to text files. HDF5 files have the advantage of being able to store multiple arrays in a single file, making them easier to transfer between machines. The Python package “DP-Data” (see Sec. III E) can generate these files from the output of an electronic calculation package.

#### 5. Model visualization

DeePMD-kit supports most of the visualization features offered by TensorBoard,^125^ such as tracking and visualizing metrics, viewing the model graph, histograms of tensors, summaries of trainable variables, and debugging profiles.

### D. Extensibility

#### 1. Application programming interface and third-party software

DeePMD-kit offers various APIs, including the Python, C++, C, and header-only C++ API, as well as a command-line interface (CLI), as shown in Fig. 2. These APIs are primarily used for inference by developers and high-level users in different situations. Sphinx^142^ generates the API details in the documentation.

These APIs can be easily accessed by various third-party software. The Python API, for instance, is utilized by third-party Python packages, such as Atomic Simulation Environment (ASE),^148^ MAGUS,^149^ and DP-Data (see Sec. III E). The C++, C, or header-only C++ API has also been integrated into several third-party MD packages, such as LAMMPS,^140,150^ i-PI,^151^ GROMACS,^152^ AMBER,^52,54,141^ OpenMM,^153,154^ and ABACUS.^155^ Moreover, the CLI is called by various third-party workflow packages, such as DP-GEN^107^ and MLatom.^34^ While the ASE calculator, the LAMMPS plugin, the i-PI driver, and the GROMACS patch are developed within the DeePMD-kit code, others are distributed separately. By integrating these APIs into their programs, researchers can perform simulations and minimization, without being restricted by DeePMD-kit’s software features.^72,75,83,156^ Additionally, they can combine DP models with other potentials outside the DeePMD-kit package if necessary.^52,72,157^

Molecular dynamics, a primary application for DP models, is facilitated by several third-party packages that interface with the DeePMD-kit package, offering a wide range of supported features:

• LAMMPS^140^ is seamlessly integrated with the DeePMD-kit through a dedicated plugin developed within the DeePMD-kit project. This plugin supports MPI, as discussed in Sec. III B 2, and provides essential functionalities, such as force calculations. Additionally, it enables on-the-fly computation of model deviation, as shown in Eqs. (68)–(72), during concurrent learning. The plugin can obtain atomic and frame-specific parameters in Eq. (38) from various sources, including constants, electronic temperatures calculated by LAMMPS, or any compute style from LAMMPS. LAMMPS also supports calculating classical point charges’ long-range (Coulomb) interaction using the Ewald summation and the fast algorithm particle–particle particle–mesh Ewald (PPPM). The k-space part of these methods, involving the Fourier space transformation of Gaussian charge distributions to compute the Coulomb interaction^158^ [as shown in Eq. (50)], is utilized by the DPLR method to handle the long-range interaction.
• i-PI^151^ is integrated with the DeePMD-kit through a dedicated driver provided within the DeePMD-kit project. The driver enables the path integral molecular dynamics (PIMD) driven by the i-PI engine and is compatible with the MolSSI Driver Interface (MDI) package^159^ and a similar interface in the ASE package.^148^ However, the communication between the i-PI driver and the engine relies on UNIX-domain sockets or the network interface, which can limit performance. To overcome this limitation, developers have incorporated PIMD features into the LAMMPS package, allowing for seamless integration with the DeePMD-kit.
• AMBER^141^ is integrated with the DeePMD-kit package through the customized source code.^54^ The AMBER/DeePMD-kit interface allows for effective QM/MM + DPRc simulations using the DPRc model.^52^ The interface extends beyond QM/QM interactions and includes a range correction for QM/MM interactions. The DeePMD-kit package only infers the selected QM region (assigned by an AMBER mask) and its MM buffer within the cutoff radius of the QM region. Like the LAMMPS integration, this interface supports MPI, as discussed in Sec. III B 2, and allows for on-the-fly computation of model deviation during concurrent learning. The AMBER/DeePMD-kit interface also enables alchemical free energy simulations to be performed, leveraging AMBER’s GPU-accelerated free energy engine^120^ and new features^160–162^ for MM transformations and using indirect MM → QM/Δ-MLP methods^163^ to correct the end states to the higher level.
• OpenMM,^153^ a widely adopted molecular dynamics engine, integrates with the DeePMD-kit through an OpenMM plugin. This plugin enables standard molecular dynamics simulations with DP models and supports hybrid DP/MM-type simulations. In hybrid simulations, the system can be simulated with a fixed DP region or adaptively changing regions during the simulation.^154^
• GROMACS^152^ is integrated with the DeePMD-kit through a patch to GROMACS. The patch enables DP/MM simulations by assigning the atom types inferred by DeePMD-kit.
• ABACUS^155^ supports the C and C++ interfaces provided by DeePMD-kit. In addition, ABACUS supports various molecular dynamics based on different methods, such as classical molecular dynamics using LJ pair potential and first-principles molecular dynamics based on methods such as Kohn-Sham density functional theory (KSDFT), stochastic density functional theory (SDFT), and orbital-free density functional theory (OFDFT). The possibility of combining the DeePMD-kit with these methods requires further exploration.

These integrations and interfaces with existing packages offer researchers the flexibility to utilize the DeePMD-kit in conjunction with other powerful tools, enhancing the capabilities of molecular dynamics simulations.

#### 2. Customized plugins

DeePMD-kit is built with an object-oriented design, and each component discussed in Sec. II corresponds to a Python class. One of the advantages of this design is the availability of a plugin system for these components. With this plugin system, developers can create and incorporate their customized components, without having to modify the DeePMD-kit package. This approach expedites the realization of their ideas. Moreover, the plugin system facilitates the addition of new components within the DeePMD-kit package itself.

### E. DeepModeling community

DeePMD-kit is a free and open-source software licensed under the LGPL-3.0 license, enabling developers to modify and incorporate DeePMD-kit into their own packages. Serving as the core, DeePMD-kit led to the formation of an open-source community named DeepModeling in 2021, which manages open-source packages for scientific computing. Since then, numerous open-source packages for scientific computing have either been created or joined the DeepModeling community, such as DP-GEN,^107^ DeePKS-kit,^164^ DMFF,^165^ ABACUS,^155^ DeePH,^166^ and DeepFlame,^167^ among others, whether directly or indirectly related to DeePMD-kit. The DeepModeling packages that are related to DeePMD-kit are listed as follows.

1. Deep Potential GENerator (DP-GEN)^107^ is a package that implements the concurrent learning procedure^103^ and is capable of generating uniformly accurate DP models with minimal human intervention and computational cost. DP-GEN2 is the next generation of this package, built on the workflow platform Dflow.
2. Deep Potential Thermodynamic Integration (DP-Ti) is a Python package that enables users to calculate free energy, perform thermodynamic integration, and determine pressure-temperature phase diagrams for materials with DP models.
3. DP-Data is a Python package that helps users convert atomistic data between different formats and calculate atomistic data through electronic calculation and MLP packages. It can be used to generate training data files for DeePMD-kit and visualize structures via 3Dmol.js.^168^ The package supports a plugin system and is compatible with ASE,^148^ allowing it to support any data format without being limited by the package’s code.
4. DP-Dispatcher is a Python package used to generate input scripts for high-performance computing (HPC) schedulers, submit them to HPC systems, and monitor their progress until completion. It was originally developed as part of the DP-GEN package,^107^ but has since become an independent package that serves other packages.
5. DArgs is a Python package that manages and filters user input arguments. It provides a Sphinx^142^ extension to generate documentation for arguments.
6. DP-GUI is a web-based graphical user interface (GUI) built with the Vue.js framework.^169^ It allows users to fill in arguments interactively on a web page and save them to a JSON^145^ file. DArgs is used to provide details and documentation of arguments in the GUI.

## IV. EXAMPLE APPLICATION: MOLECULAR DYNAMICS

**FIG. 3.**
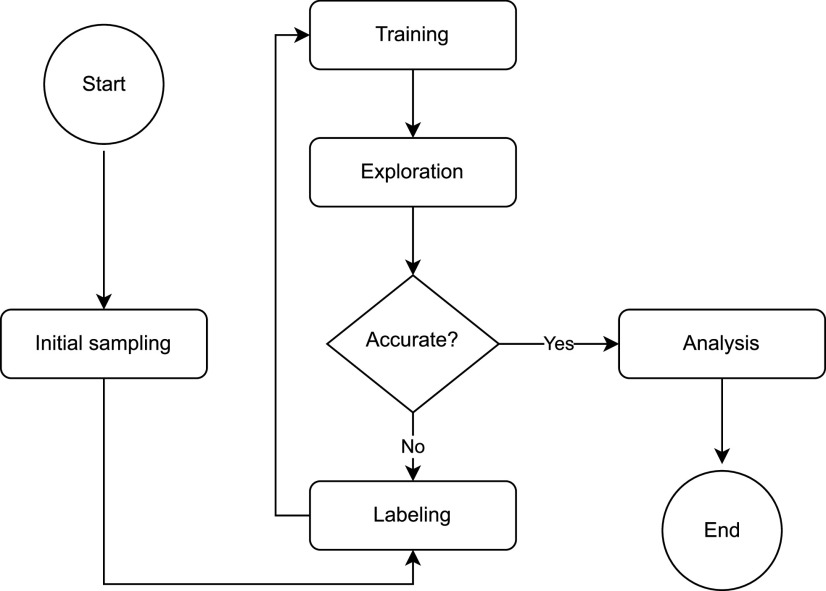
The general workflow of performing deep potential molecular dynamics in the manner of concurrent learning.

This section introduces a general workflow for performing deep potential molecular dynamics using concurrent learning^170^ from scratch, as depicted in Fig. 3. The target simulation can encompass various conditions, such as temperature, pressure, and classical or path-integral dynamics, with or without enhanced sampling methods, in equilibrium or non-equilibrium states, and at different scales and time scales. It is important to note that this section does not serve as a user manual or tutorial or delve into specific systems.

The initial step involves preparing the initial dataset. This dataset is typically generated by sampling from small-scale, short-time MD simulations conducted under the same conditions as target simulations. The simulation level can vary, ranging from *ab initio*^170^ to semi-empirical^52^ or force fields,^77^ depending on the computational cost. Subsequently, these configurations are relabeled using high-accuracy *ab initio* methods.

Once the initial data are ready, the next step involves performing concurrent learning cycles, which are crucial for improving the accuracy of the target simulation. Each cycle comprises three steps: training, exploration, and labeling. In the training step, DeePMD-kit trains multiple models (typically four models) using the existing target data collection with short training steps. These models can be initialized from different random seeds or from the models trained in the previous iteration. In the exploration step, one of the models is employed to perform the target simulation and sample the configurational space. If the target simulation involves a non-equilibrium process, the simulation time can gradually increase with concurrent learning cycles. Configurations (or a subset of atoms within the configurations to reduce computational cost^77^) are randomly selected from configurations that satisfy the following condition:

$$\left\{R_{n}|n\in I_{\text{cand}},I_{\text{cand}}=\left\{n|\theta_{\text{low}}\leq \epsilon_{F,\max}<\theta_{\text{high}}\right\}\right\},$$ (74)

where *ϵ*_**F**,max_ was given in Sec. II C, *θ*_low_ should be set to a value higher than most of *ϵ*_**F**,max_ in the existing target data collection, and *θ*_high_ is typically set to a value ∼0.15 eV/Å higher than *θ*_low_. These threshold values ensure that only configurations not yet added to the target data collection will be selected. The selected configurations are labeled using consistent *ab initio* methods and added to the target data collection in the labeling step, proceeding to the next iteration.

If the ratio of accurate configurations (*ϵ*_**F**,max_ < *θ*_low_) in a simulation converges (remains unchanged in subsequent concurrent learning cycles), it can be considered as the target simulation, and the iteration can be stopped. Such a simulation trajectory can be further analyzed.

The above workflow can be executed manually or using the DP-GEN package^107^ automatically.

## V. BENCHMARKING

We performed benchmarking on various potential energy models with different descriptors on multiple datasets to show the precision and performance of descriptors developed within the DeePMD-kit package. The datasets, the models, the hardware, and the results will be described and discussed in the following Secs. V A–V C.

### A. Datasets

The datasets we used included water,^9,61^ copper (Cu),^107^ high entropy alloys (HEAs),^51,171^ OC2M subset in Open Catalyst 2020 (OC20),^115,116^ Small-Molecule/Protein Interaction Chemical Energies (SPICEs),^104^ and dipeptide subset in SPICE,^104^ as shown in Table I and listed as follows:

• The water dataset contains of 140 000 configurations collected from path-integral *ab initio* MD simulations and classical *ab initio* MD simulations for liquid water and ice. Configurations were labeled using the hybrid version of Perdew–Burke–Ernzerhof (PBE0)^172^+ Tkatchenko–Scheffler (TS) functional and projector augmented-wave (PAW) method.^173^ The energy cutoff was set to 115 Ry (1565 eV).
• The copper dataset consists of 15 366 configurations in Face Centered Cubic (FCC), Hexagonal Close Packed (HCP), and Body Centered Cubic (BCC) crystal. MD simulations sampled the configurations across a temperature range of 50–2579 K and a pressure range of 1–5 × 10^4^ Bar. The concurrent learning scheme^170^ was employed to select the critical configurations that improved the accuracy of an ensemble of models used to estimate the model prediction error. The Perdew–Burke–Ernzerhof (PBE) functional^174^ and PAW method were used with an energy cutoff of 650 eV.
• The High Entropy Alloy (HEA) dataset comprises six elements: Ta, Nb, W, Mo, V, and Al.^51,171^ These elements occupy a 2 × 2 × 2 BCC lattice consisting of 16 atoms in a random arrangement. The concentrations of Ta, Nb, W, Mo, and V encompass the entire composition space, while Al is considered an additive, with its maximum quantity being less than six. MD simulations sampled the configurations across a temperature range of 50–388.1 K and a pressure range of 1–5 × 10^4^ bars. The concurrent learning scheme^170^ was employed to select the critical configurations that improved the accuracy of an ensemble of models used to estimate the model prediction error. The dataset comprises 8160 configurations labeled by the density functional theory with PBE approximation^174^ of the exchange and correlation. The PAW method was used with an energy cutoff of 1200 eV and a k-space sampling grid size of 0.12 Å^−1^.
• The OC2M subset^116^ in the Open Catalyst 2020 (OC20) dataset takes 2 × 10^6^ configurations from the OC20 dataset^115^ and includes 57 elements. OC20 consists of 1 281 040 configurations across a wide swath of materials, surfaces, and adsorbates and is labeled by the revised PBE functional^174^ under the periodic boundary condition. The PAW method was employed with an energy cutoff of 350 eV.
• The Small-Molecule/Protein Interaction Chemical Energies (SPICE)^104^ dataset is a drug-like dataset that includes various subsets: dipeptides, solvated amino acids, PubChem molecules, DES370K dimers, DES370K monomers, and ion pairs. The dataset is composed of 1 132 808 non-period configurations labeled at the *ω*B97M-D3BJ/def2-TZVPPD level.^175,176^ It consists of 15 elements and contains charged configurations. We adopted the same method as described in Ref. 104 to consider each unique combination of element and formal charge as a different atom type.
• The dipeptide subset in SPICE^104^ comprises all possible dipeptides formed by the 20 natural amino acids and their common protonation variants. This subset contains 33 850 configurations with elements, including H, C, N, O, and S, corresponding to the amino acids.

**TABLE I.** Datasets used to benchmark.

Dataset	No. of frames	Elements	DFT level	References
Water	140 000	H, O	PBE0+TS/PAW (*E*_cutoff_ = 1565 eV)	9 and 61
Copper	15 366	Cu	PBE/PAW (*E*_cutoff_ = 650 eV)	107
HEA	8 160	Ta, Nb, W, Mo, V, Al	PBE/PAW(*E*_cutoff_ = 1200 eV)	51 and 171
OC2M	2 000 000	Ag, Al, As, Au, B, Bi, C, Ca, Cd, Cl, Co, Cr, Cs, Cu,	RPBE/PAW (*E*_cutoff_ = 350 eV)	115 and 116
		Fe, Ga, Ge, H, Hf, Hg, In, Ir, K, Mg, Mn, Mo, N, Na, Nb,		
		Ni, O, Os, P, Pb, Pd, Pt, Rb, Re, Rh, Ru, S, Sb, Sc, Se,		
		Si, Sn, Sr, Ta, Tc, Te, Ti, Tl, V, W, Y, Zn, Zr		
SPICE	1 132 808	H, Li, C, N, O, F, Na, Mg, P, S, Cl, K, Ca, Br, I	*ω*B97M-D3BJ/def2-TZVPPD	104
Dipeptides	33 850	H, C, N, O, S	*ω*B97M-D3BJ/def2-TZVPPD	104

The above datasets are representative as they contain liquids, solids, and gases, configurations in both periodic and non-periodic boundary conditions, configurations spanning a wide range of temperatures and pressures, and ions and drug-like molecules in different protonation states. The study of all these systems is essential in the field of chemical physics.

We split all the datasets into a training set containing 95% of the data and a validation set containing the remaining 5% of the data.

### B. Models and hardware

We compared various descriptors, including the local frame (loc_frame), two-body embedding full-information DeepPot-SE (se_e2_a), a hybrid descriptor with two-body embedding full- and radial-information DeepPot-SE (se_e2_a+se_e2_r), a hybrid descriptor with two-body embedding full-information and three-body embedding DeepPot-SE (se_e2_a+se_e3), and an attention-based descriptor (se_atten). In all models, we set *r*_*s*_ to 0.5 Å, *M*_<_ to 16, and *L*_*a*_ to 2, if applicable. We used (25, 50, 100) neurons for two-body embedding networks $N_{\;e,2}$, (2, 4, 8) neurons for three-body embedding networks $N_{\;e,3}$, and (240, 240, 240, 1) neurons for fitting networks $F_{0}$. In the full-information part (se_e2_a) of the hybrid descriptor with two-body embedding full-information and radius-information DeepPot-SE (se_e2_a+se_e2_r) and the two-body embedding part (se_e2_a) of the hybrid descriptor with two-body full-information and three-body DeepPot-SE (se_e2_a+se_e3), we set *r*_*c*_ to 4 Å. For the OC2M system, we set *r*_*c*_ to 9 Å, while under other situations, we set *r*_*c*_ to 6 Å.

We trained each model for a fixed number of steps (1 000 000 for water, Cu, and dipeptides, 16 000 000 for HEA, and 10 000 000 for OC2M and SPICE) using neural networks in double floating precision (FP64) and single floating precision (FP32) separately. We used the LAMMPS package^140^ to perform MD simulations for water, Cu, and HEA with as many atoms as possible. We compared the performance of compressed models with that of the original model where applicable.^108^ The platforms used to benchmark performance included 128-core AMD EPYC 7742, NVIDIA GeForce RTX 3080 Ti (12 GB), NVIDIA Tesla V100 (40 GB), NVIDIA Tesla A100 (80 GB), AMD Instinct MI250, and Xilinx Virtex Ultrascale + VU9P FPGA for NVNMD only.^110^ We note that currently, the model compression feature only supports se_e2_a, se_e2_r, and se_e3 descriptors, and NVNMD only supports regular se_e2_a for systems with no more than four chemical species in FP64 precision. The model compression feature for se_atten is under development.

It is important to note that these models are designed for the purpose of comparing different descriptors and floating-point number precisions supported by the package under the same conditions, with the aim of recommending the best model to use. However, it should be emphasized that the number of training steps is limited, and hyperparameters, such as the number of neurons in neural networks, are not tuned for any specific system. Therefore, it is not advisable to utilize these models for production purposes, and it would be meaningless to compare them with the well-established models reported in other references, Refs. 9, 51, 107, and 104.

Furthermore, it is not recommended to compare these models with models produced by other packages, as it can be challenging to establish a fair comparison. For instance, ensuring that hyperparameters in different models are precisely the same or making all models consume the same computational resources across different packages is not straightforward.

### C. Results and discussion

We present the validation errors of different models in Table II, the training and MD performance on various platforms in Tables III and IV, as well as the maximum number of atoms that a platform can simulate in Table V. None of the models outperforms the others in terms of accuracy for all datasets. The non-smooth local frame descriptor achieves the best accuracy for the water system, with an energy RMSE of 0.689 meV/atom and a force RMSE of 39.2 meV/Å. Moreover, this model exhibits the fastest computing performance among all models on CPUs, although it has not yet been implemented on GPUs, as shown in Appendix B. The local frame descriptor, despite having higher accuracy in some cases, has limitations that hinder its widespread applicability. One such limitation is that it is not smooth. Additionally, this descriptor does not perform well for the copper system, which was collected over a wide range of temperatures and pressures.^107^ Another limitation is that it requires all systems to have similar chemical species to build the local frame, which makes it challenging to apply in datasets, such as HEA, OC2M, dipeptides, and SPICE.

**TABLE II.** Root mean square errors (RMSEs) in the energy per atom (E, meV/atom) and forces (**F**, meV/Å) for water, Cu, HEA, OC2M, dipeptides, and SPICE validation sets. The boldfaced values denote the best model in an indicator.

		loc_frame		se_e2_a		se_e2_a+se_e2_r		se_e2_a+se_e3		se_atten	
System	Indicator	FP64	FP32	FP64	FP32	FP64	FP32	FP64	FP32	FP64	FP32
Water	E RMSE	0.7	**0.7**	1.0	1.0	0.9	1.0	1.0	1.0	1.5	1.2
	**F** RMSE	40.0	**39.2**	49.0	48.4	48.6	50.0	46.5	45.9	44.4	42.3
Cu	E RMSE	12.7	19.2	3.0	2.8	4.8	4.9	**2.5**	2.6	3.2	3.6
	**F** RMSE	84.7	105	17.7	17.9	21.4	22.0	16.8	**16.6**	16.9	16.9
HEA	E RMSE	⋯	⋯	15.4	15.3	13.5	14.5	12.1	17.2	**5.5**	6.4
	**F** RMSE	⋯	⋯	134	137	163	158	136	180	**90.7**	98.3
OC2M	E RMSE	⋯	⋯	⋯	⋯	⋯	⋯	⋯	⋯	15.1	**14.3**
	**F** RMSE	⋯	⋯	⋯	⋯	⋯	⋯	⋯	⋯	155	**148**
Dipeptides	E RMSE	⋯	⋯	**9.5**	9.6	12.5	11.7	14.9	16.8	12.8	12.7
	**F** RMSE	⋯	⋯	97.9	97.7	98.6	101	160	222	99.7	**96.7**
SPICE	E RMSE	⋯	⋯	⋯	⋯	⋯	⋯	⋯	⋯	80.9	**78.3**
	**F** RMSE	⋯	⋯	⋯	⋯	⋯	⋯	⋯	⋯	**233**	234

**TABLE III.** Training performance (ms/step) for water, Cu, HEA, OC2M, dipeptides, and SPICE systems. “FP64” means double floating precision, “FP32” means single floating precision, and “FP64c” and “FP32c” mean the compressed training^109^ for double and single floating precision, respectively. “EPYC” performed on 128 AMD EPYC 7742 cores, “3080 Ti” performed on an NVIDIA GeForce RTX 3080 Ti card, “V100” performed on an NVIDIA Tesla V100 card, “A100” performed on an NVIDIA Tesla A100 card, and “MI250” performed on an AMD Instinct MI250 Graphics Compute Die (GCD).

		loc_frame		se_e2_a				se_e2_a+se_e2_r				se_e2_a+se_e3				se_atten	
System	Hardware	FP64	FP32	FP64	FP32	FP64c	FP32c	FP64	FP32	FP64c	FP32c	FP64	FP32	FP64c	FP32c	FP64	FP32
Water	EPYC	14.7	9.20	97.3	45.0	28.4	16.2	63.7	32.5	29.9	15.4	141	85.2	34.0	20.6	1210	383
	3080 Ti	7.00	4.80	24.6	10.3	9.70	6.40	26.3	11.6	12.0	8.20	52.8	17.2	16.3	6.80	199	26.9
	V100	7.90	8.50	11.1	8.20	5.90	4.80	13.6	10.9	6.90	6.40	23.5	14.0	8.60	7.30	69.6	31.7
	A100	10.7	10.0	8.20	9.30	4.90	5.70	14.5	10.8	7.80	6.30	24.5	12.0	7.50	7.20	30.8	21.2
	MI250	11.7	10.9	20.3	13.1	7.70	7.00	27.3	19.7	11.5	10.9	278	27.7	12.8	11.2	125	31.7
Cu	EPYC	4.90	3.30	33.7	12.8	8.00	5.40	19.9	10.0	10.5	5.30	45.5	24.2	9.10	6.50	226	89.1
	3080 Ti	3.20	2.20	6.50	5.10	4.60	3.90	8.70	6.30	5.90	3.40	11.8	4.80	7.20	5.70	36.8	8.80
	V100	3.20	3.80	4.20	4.80	3.20	3.70	6.50	5.30	5.50	4.10	7.90	5.60	6.00	5.80	15.6	11.9
	A100	4.00	3.90	3.80	3.70	3.10	3.00	5.40	5.30	4.10	4.10	8.00	5.60	4.80	4.60	11.6	11.2
	MI250	4.80	4.90	6.90	6.40	5.10	5.00	9.10	9.40	7.40	7.00	49.9	10.1	8.00	7.30	23.6	18.6
HEA	EPYC	⋯	⋯	53.4	30.5	19.4	12.2	52.3	29.3	27.7	16.7	83.7	51.1	26.6	15.7	159	60.1
	3080 Ti	⋯	⋯	38.4	25.2	11.2	9.10	71.4	41.8	16.3	12.7	93.6	41.0	19.7	15.0	35.9	9.10
	V100	⋯	⋯	33.2	29.8	11.8	11.1	63.2	47.4	17.5	16.5	65.5	49.6	27.4	18.7	15.6	11.9
	A100	⋯	⋯	30.5	28.6	10.9	10.4	51.6	67.4	16.9	21.2	61.7	52.9	18.6	18.8	11.7	11.5
	MI250	⋯	⋯	48.8	42.7	18.5	18.0	72.3	69.3	28.7	27.3	134	88.4	32.7	32.3	21.6	19.5
OC2M	EPYC	⋯	⋯	⋯	⋯	⋯	⋯	⋯	⋯	⋯	⋯	⋯	⋯	⋯	⋯	2070	625
	3080 Ti	⋯	⋯	⋯	⋯	⋯	⋯	⋯	⋯	⋯	⋯	⋯	⋯	⋯	⋯	352	46.0
	V100	⋯	⋯	⋯	⋯	⋯	⋯	⋯	⋯	⋯	⋯	⋯	⋯	⋯	⋯	120	52.8
	A100	⋯	⋯	⋯	⋯	⋯	⋯	⋯	⋯	⋯	⋯	⋯	⋯	⋯	⋯	51.4	30.9
	MI250	⋯	⋯	⋯	⋯	⋯	⋯	⋯	⋯	⋯	⋯	⋯	⋯	⋯	⋯	171	55.7
Dipeptides	EPYC	⋯	⋯	49.7	30.5	21.2	19.4	52.0	35.3	30.1	21.2	89.5	61.1	35.0	21.2	214	91.5
	3080 Ti	⋯	⋯	54.8	39.5	17.3	11.3	90.0	64.3	19.0	15.3	131	67.7	25.4	19.2	26.1	12.0
	V100	⋯	⋯	54.1	52.6	14.8	14.8	88.0	84.3	20.5	21.7	96.2	103	30.1	30.8	14.3	10.6
	A100	⋯	⋯	50.2	50.8	14.3	14.3	89.0	75.9	20.7	19.9	91.1	82.7	26.6	26.7	13.2	11.1
	MI250	⋯	⋯	66.2	67.8	23.1	22.9	117	112	35.0	32.4	155	129	45.9	44.9	19.6	16.8
SPICE	EPYC	⋯	⋯	⋯	⋯	⋯	⋯	⋯	⋯	⋯	⋯	⋯	⋯	⋯	⋯	244	98.0
	3080 Ti	⋯	⋯	⋯	⋯	⋯	⋯	⋯	⋯	⋯	⋯	⋯	⋯	⋯	⋯	35.4	15.3
	V100	⋯	⋯	⋯	⋯	⋯	⋯	⋯	⋯	⋯	⋯	⋯	⋯	⋯	⋯	17.3	15.9
	A100	⋯	⋯	⋯	⋯	⋯	⋯	⋯	⋯	⋯	⋯	⋯	⋯	⋯	⋯	11.9	12.2
	MI250	⋯	⋯	⋯	⋯	⋯	⋯	⋯	⋯	⋯	⋯	⋯	⋯	⋯	⋯	29.0	24.1

**TABLE IV.** MD performance (*μ*s/step/atom) for water, Cu, and HEA systems. “FP64” means double floating precision, “FP32” means single floating precision, and “FP64c” and “FP32c” mean the compressed model^109^ for double and single floating precision, respectively. “EPYC” performed on 128 AMD EPYC 7742 cores, “3080 Ti” performed on an NVIDIA GeForce RTX 3080 Ti card, “V100” performed on an NVIDIA Tesla V100 card, “A100” performed on an NVIDIA Tesla A100 card, “MI250” performed on an AMD Instinct MI250 Graphics Compute Die (GCD), and “VU9P” performed NVNMD^110^ on a Xilinx Virtex Ultrascale+ VU9P FPGA board.

		loc_frame		se_e2_a				se_e2_a+se_e2_r				se_e2_a+se_e3				se_atten	
System	Hardware	FP64	FP32	FP64	FP32	FP64c	FP32c	FP64	FP32	FP64c	FP32c	FP64	FP32	FP64c	FP32c	FP64	FP32
Water	EPYC	1.25	0.699	19.3	8.73	3.89	2.61	8.33	3.43	3.78	1.86	37.2	15.1	5.04	3.63	221	83.8
	3080 Ti	12.9	8.63	29.0	4.21	9.71	1.73	20.8	3.43	9.06	1.99	69.5	10.5	18.5	2.89	294	32.3
	V100	16.1	16.8	8.25	4.59	1.94	1.51	6.21	3.53	2.22	1.62	22.2	11.3	3.31	2.41	91.2	37.2
	A100	35.7	33.9	4.37	3.01	1.56	1.42	4.11	2.44	2.07	1.53	12.5	7.17	2.64	2.25	35.6	22.4
	MI250	40.2	39.6	7.74	3.96	1.74	1.41	6.03	3.20	2.00	1.54	30.5	18.8	3.51	2.64	55.0	30.2
	VU9P	⋯	⋯	0.306	⋯	⋯	⋯	⋯	⋯	⋯	⋯	⋯	⋯	⋯	⋯	⋯	⋯
Cu	EPYC	1.14	0.702	22.2	9.38	3.43	2.04	11.9	5.28	3.09	1.56	47.9	19.5	4.20	2.73	200	62.1
	3080 Ti	14.9	8.98	30.5	4.18	8.52	1.51	18.8	3.15	7.98	1.81	74.6	11.2	14.7	2.32	294	33.0
	V100	15.7	15.7	8.73	4.81	1.56	1.27	5.71	3.18	1.84	1.38	24.3	12.2	2.60	1.83	91.1	37.3
	A100	36.9	36.9	4.41	2.65	1.36	1.15	3.35	2.15	1.63	1.42	13.5	7.49	2.15	1.78	36.2	21.0
	MI250	39.0	39.1	8.27	4.13	1.37	1.21	5.62	2.98	1.59	1.35	26.9	12.6	2.56	2.00	55.4	29.5
	VU9P	⋯	⋯	0.310	⋯	⋯	⋯	⋯	⋯	⋯	⋯	⋯	⋯	⋯	⋯	⋯	⋯
HEA	EPYC	⋯	⋯	32.8	13.0	7.04	4.58	15.3	7.64	6.83	3.80	81.0	33.4	8.56	5.68	156	45.9
	3080 Ti	⋯	⋯	65.3	9.72	10.5	2.51	36.1	6.83	11.9	3.24	171	24.9	29.6	5.37	290	32.8
	V100	⋯	⋯	20.1	10.9	2.88	2.39	12.3	6.86	12.3	2.85	55.2	28.4	9.42	5.47	91.2	37.4
	A100	⋯	⋯	10.4	6.09	2.13	1.83	7.25	5.48	2.98	2.83	30.1	17.1	4.21	4.22	35.0	20.0
	MI250	⋯	⋯	20.1	11.6	4.57	4.22	16.2	12.0	7.01	6.44	76.0	44.9	9.09	7.61	55.7	30.5

**TABLE V.** The maximum number of atoms (10^3^) that a GPU card can simulate for water, Cu, and HEA systems. “FP64” means double floating precision, “FP32” means single floating precision, and “FP64c” and “FP32c” mean the compressed model^109^ for double and single floating precision, respectively. “3080 Ti” performed on an NVIDIA GeForce RTX 3080 Ti card (12 GB), “V100” performed on an NVIDIA Tesla V100 card (40 GB), and “A100” performed on an NVIDIA Tesla A100 card (80 GB).^a^

		se_e2_a				se_e2_a+se_e2_r				se_e2_a+se_e3				se_atten	
System	Hardware	FP64	FP32	FP64c	FP32c	FP64	FP32	FP64c	FP32c	FP64	FP32	FP64c	FP32c	FP64	FP32
Water	3080 Ti	27	51	127	141	44	74	94	128	10	21	86	95	3	7
	V100	73	135	415	493	114	196	274	430	28	55	250	265	9	19
	A100	189	332	987	1128	288	488	651	900	76	147	618	736	22	49
Cu	3080 Ti	18	35	214	202	40	77	125	151	7	14	83	144	3	7
	V100	54	106	606	635	122	183	337	461	19	39	271	330	9	19
	A100^b^	141	244	1534	1615	286	453	706	1074	50	99	697	867	22	49
HEA	3080 Ti	11	19	60	69	21	33	47	59	5	9	42	52	3	7
	V100	30	53	175	184	57	87	131	166	13	25	117	142	9	19
	A100	76	132	447	468	140	218	323	408	35	66	292	365	22	48

^a^ The results on the MI250 card are not reported since the ROCm Toolkit reported a segmentation fault when the number of atoms increased.

^b^ Two MPI ranks were used since integer overflow occurred in TensorFlow when the number of elements in an operator exceeded 2^31^.

On the other hand, the DeepPot-SE descriptor offers greater generalization in terms of both accuracy and performance. The compressed models are 1–10× faster than the original for training and inference, and the NVNMD is 50–100× faster than the regular MD, both of which demonstrate impressive computational performance. It is expected that the MD performance of the uncompressed water se_e2_a FP64 model (8.25 *µ*s/step/atom on a single V100 card) is close to the MD performance reported in Ref. 41 (8.19 *µ*s/step/atom per V100 card), and the MD performance of the compressed model (1.94 *µ*s/step/atom) is about 3× faster in this case. In addition, the compressed model can simulate 6× atoms in a single card compared to the uncompressed model. The three-body embedding descriptor theoretically contains more information than the two-body embedding descriptor and is expected to be more accurate but slower. While this is true for the water and copper systems, the expected order of accuracy is not clearly observed for the HEA and dipeptides datasets. Further research is required to determine the reason for this discrepancy, but it is likely due to the loss not converging within the same training steps when more chemical species result in more trainable parameters. Furthermore, the performance on these two datasets slows down as there are more neural networks.

The attention-based models with the type embedding exhibit better accuracy for the HEA system and equivalent accuracy for the dipeptide system. These models also have the advantage of faster training on GPUs, with equivalent accuracy for these two systems, by reducing the number of neural networks. However, this advantage is not observed on CPUs or MD simulations, as attention layers are computationally expensive, which calls for future improvements. Furthermore, when there are many chemical species, the attention-based descriptor requires less CPU or GPU memory than other models since it has fewer neural networks. This feature makes it possible to apply to the OC2M dataset with over 60 species and the SPICE dataset with about 20 species.

It is noteworthy that in nearly all systems, FP32 is 0.5 to 2× faster than FP64 and demonstrates similar validation errors. In this case, since we only apply FP32 into neural networks but keep precision in other components, the model precision is also well-known as “mixed precision.” This result is consistent with the fact that mixed precision has been widely adopted in other packages.^38,140^ Therefore, FP32 should be widely adopted in most applications. Moreover, FP32 enables high performance on hardware with poor FP64 performance, such as consumer GPUs or CPUs.

## VI. SUMMARY

DeePMD-kit is a powerful and versatile community-developed open-source software package for molecular dynamics (MD) simulations using machine learning potentials (MLPs). Its excellent performance, usability, and extensibility have made it a popular choice for researchers in various fields. DeePMD-kit is licensed under the LGPL-3.0 license, which allows anyone to use, modify, and extend the software freely. Thanks to its well-designed code architecture, DeePMD-kit is highly customizable and can be easily extended in various aspects. The models are organized as Python modules in an object-oriented design and saved into the computing graphs, making it easier to add new models. The computing graph is composed of TensorFlow and customized operators, making it easier to optimize the package for a particular hardware architecture and certain operators. The package also has rich and flexible APIs, making it easier to integrate with other molecular simulation packages. DeePMD-kit is open to contributions from researchers in computational science, and we hope that the community will continue to develop and enhance its features in the future.

## Data Availability

DeePMD-kit is openly hosted at the GitHub repository: https://github.com/deepmodeling/deepmd-kit. The datasets, the models, the simulation systems, and the benchmarking scripts used in this study can be downloaded from the GitHub repository: https://github.com/deepmodeling-activity/deepmd-kit-v2-paper. Other data that support the findings of this study are available from the corresponding author upon reasonable request.

## APPENDIX A: FIFTH-ORDER POLYNOMIAL INTERPOLATION

Define a piecewise-defined function *f*(*x*), where *f*(*x*) is a fifth-order polynomial in the range [0, 1) and is a constant in other intervals,

$$f\left(x\right)=\left\{\begin{array}{cc} 1,\; & x<0,\\ ax^{5}+bx^{4}+cx^{3}+dx^{2}+ex+f,\; & 0\leq x<1,\\ 0,\; & x\geq 1. \end{array}\right.$$ (A1)

Let *f*(*x*), its first-order derivative *f*′(*x*), and its second-order derivative *f*^″^(*x*) be continuous at *x* = 0 and *x* = 1,

$$\left\{\begin{array}{c} \lim_{x\to 0^{-}}f\left(x\right)=\lim_{x\to 0^{+}}f\left(x\right),\;\\ \lim_{x\to 0^{-}}f^{\prime }\left(x\right)=\lim_{x\to 0^{+}}f^{\prime }\left(x\right),\;\\ \lim_{x\to 0^{-}}f^{\prime \prime }\left(x\right)=\lim_{x\to 0^{+}}f^{\prime \prime }\left(x\right),\;\\ \lim_{x\to 1^{-}}f\left(x\right)=\lim_{x\to 1^{+}}f\left(x\right),\;\\ \lim_{x\to 1^{-}}f^{\prime }\left(x\right)=\lim_{x\to 1^{+}}f^{\prime }\left(x\right),\;\\ \lim_{x\to 1^{-}}f^{\prime \prime }\left(x\right)=\lim_{x\to 1^{+}}f^{\prime \prime }\left(x\right).\; \end{array}\right.$$ (A2)

Solve Eq. (A2), and the solution is

a=−6,b=15,c=−10,d=0,e=0,f=1. (A3)

The final forum of *f*(*x*) is

$$f\left(x\right)=\left\{\begin{array}{cc} 1,\; & x<0,\\ -6x^{5}+15x^{4}-10x^{3}+1,\; & 0\leq x<1,\\ 0,\; & x\geq 1. \end{array}\right.$$ (A4)

## APPENDIX B: FEATURES WITHOUT GPU SUPPORT

At present, the following features do not have GPU support:

• The local frame descriptor in Eqs. (5)–(10).
• Interpolation with a pairwise potential in Eqs. (52)–(55).
• Calculating the maximum number of neighbors *N*_*c*_ within the cutoff radius from the given data.
• Model deviation in Eqs. (68)–(72).
• All NVNMD-specific features.
• The KSpace solver for DPLR in the LAMMPS plugin.
